# Tetraspanin 1 promotes epithelial-to-mesenchymal transition and metastasis of cholangiocarcinoma via PI3K/AKT signaling

**DOI:** 10.1186/s13046-018-0969-y

**Published:** 2018-12-04

**Authors:** Yan Wang, Yingjian Liang, Guangchao Yang, Yaliang Lan, Jihua Han, Jiabei Wang, Dalong Yin, Ruipeng Song, Tongsen Zheng, Shugeng Zhang, Shangha Pan, Xirui Liu, Mingxi Zhu, Yao Liu, Yifeng Cui, Fanzheng Meng, Bo Zhang, Shuhang Liang, Hongrui Guo, Yufeng Liu, Md Khaled Hassan, Lianxin Liu

**Affiliations:** 1 Department of Hepatic Surgery, The First Affiliated Hospital of Harbin Medical University, Key Laboratory of Hepatosplenic Surgery, Ministry of Education, Harbin, Heilongjiang China; 2 Department of Gastrointestinal Medical Oncology, The Affiliated Tumor Hospital of Harbin Medical University, Harbin, Heilongjiang China; 3 Department of Pharmacology (the State-Province Key Laboratories of Biomedicine-Pharmaceutics of China, Key Laboratory of Cardiovascular Research, Ministry of Education), Harbin Medical University, Harbin, China

**Keywords:** Cholangiocarcinoma, Tetraspanin 1, Epithelial-to-mesenchymal transition, MicroRNA-194-5p, Integrin α6β1

## Abstract

**Background:**

Numerous studies have demonstrated that tetraspanin 1 (TSPAN1), a transmembrane protein, functions as an oncoprotein in many cancer types. However, its role and underlying molecular mechanism in cholangiocarcinoma (CCA) progression remain unclear.

**Methods:**

In the present study, the expression of TSPAN1 in human CCA and adjacent nontumor tissues was examined using real-time PCR, western blot and immunohistochemistry. The effect of TSPAN1 on proliferation and metastasis was evaluated by functional assays both in vitro and in vivo. A luciferase reporter assay was performed to investigate the interaction between microRNA-194-5p (miR-194-5p) and TSPAN1 3′-untranslated region. Co-immunoprecipitation (co-IP) was used to confirm the interaction between TSPAN1 protein and integrin α6β1 and western blot was used to explore TSPAN1 mechanism.

**Results:**

We found that TSPAN1 was frequently upregulated in CCA and high levels of TSPAN1 correlated with TNM stage, especially metastasis in CCA. TSPAN1 overexpression promoted CCA growth, metastasis, and induced epithelial-to-mesenchymal transition (EMT), while its silencing had the opposite effect both in vitro and in vivo. To explore the differential expression of TSPAN1, we screened miR-194-5p as the upstream regulator of TSPAN1. A combination of high-level TSPAN1 and low-level miR-194-5p predicted poor prognosis in patients with CCA. Furthermore, in accordance with the functional characteristics of the TSPAN superfamily, we proved that TSPAN1 interacted with integrin α6β1 to amplify the phosphoinositide-3-kinase (PI3K)/AKT/glycogen synthase kinase (GSK)-3β/Snail family transcriptional repressor (Snail)/phosphatase and tensin homolog (PTEN) feedback loop.

**Conclusion:**

The results indicate that TSPAN1 could be a potential therapeutic target for CCA.

**Electronic supplementary material:**

The online version of this article (10.1186/s13046-018-0969-y) contains supplementary material, which is available to authorized users.

## Background

Human cholangiocarcinoma (CCA), an epithelial cell malignancy arising from varying locations in the biliary tree, is the second most common primary hepatic cancer worldwide [1]. Currently, surgical resection is the only curative treatment. However, the resectability and curability remain low. Therefore, it is critical to elucidate the molecular mechanisms regulating CCA tumor progression to find potential therapeutic strategies.

Tetraspanin 1 (TSPAN1) is a member of the TSPAN family of proteins whose important feature is their ability to aggregate with one another or various other transmembrane receptors, to become TSPAN-enriched microdomains (TEMs). TEMs are essential in determining the fundamental biological activities such as cell adhesion, proliferation and cell motility [2]. Recently, TSPAN1 was reported to accelerate many kinds of cancer progression, especially digestive malignancies such as hepatocellular carcinoma (HCC), pancreatic, gastric, colorectal, and esophageal cancers [3–7], and some other non-digestive cancers such as osteosarcoma and cervical cancer [8, 9]. In these studies, TSPAN1 was treated as an oncogene promoting cancer process. However, its exact mechanism is unknown. Therefore, for the first time, we previously demonstrated that TSPAN1 was a critical promoter in human CCA and explored its mechanism. Coincidently, Subrungruanga et al. [10] conducted a whole-transcript expression array study using microarray profiling of 15 pairs of intrahepatic CCA tumors and corresponding normal liver tissue samples, and TSPAN1 was one of the 42 upregulated genes in intrahepatic CCA.

MicroRNAs (miRNAs), are small non-coded strands of RNAs that regulate post-transcriptional gene expression and silence an extensive range of target genes. Oncogenes or tumor suppressor genes regulated by miRNAs have a wide range of functions in cancer, indicating that they could act as therapeutic targets or biomarkers for the diagnosis of cancer [11]. To date, miR-194-5p has been studied in several distinct cancer types such as HCC, gallbladder cancer, glioblastoma, and acute myeloid leukemia [12–16]. In our study, for the first time, we investigated the tumor suppressor role of miR-194-5p in CCA.

Accumulating evidence shows that epithelial-mesenchymal transition (EMT) has a critical role in the dissemination of malignant oncocytes during CCA progression [17, 18]. Integrins are a family of cell adhesion receptors that are transmembrane α-β heterodimers, and 18α and 8β subunits have been currently identified [19]. This family of proteins recognizes extracellular matrix (ECM) ligands, cell surface molecules, and growth factors to activate further several intracellular signaling pathways, which regulate different pathological progress including EMT [20]. TSPANs are peculiar molecular scaffolds that distribute proteins into highly organized microdomains, and the integrin family is one of the most prominent classes of adhesion receptors that bind TSPANs. In this study, we screened several integrins, which could interact directly with TSPAN1 protein and identified integrin α6β1. Lastly, we demonstrated that TSPAN1 promoted integrin α6β1 downstream phosphoinositide-3-kinase (PI3K)/AKT/glycogen synthase kinase (GSK)-3β/snail family transcriptional repressor (Snail)/phosphatase and tensin homolog (PTEN) feedback loop, which is responsible for the EMT of CCA.

## Methods

### Cell lines and culture

The HIBEpiC normal human biliary cell line was obtained from ScienCell Research Laboratories (Carlsbad, CA, USA). The L02 immortalized normal liver cell line was purchased from the Institute of Biochemistry and Cell Biology, Chinese Academy of Science, China. The HuCCT1 cell line was kindly provided by the Cancer Cell Repository of the Tohoku University in Japan. CCLP1 and KMBC cell lines were purchased from BeNa Culture Collection (Beijing, China). RBE and HCCC9810 cell lines were purchased from Shanghai Bioleaf Biotech Corporation (Shanghai, China). Huh28 was purchased from Accegen Biotechnology Corporation (Fairfield, NJ, USA). Huh28, RBE, HCCC9810 and HuCCT1 were authenticated using Short Tandem Repeat (STR) analysis. All cell lines were cultured in Dulbecco’s modified Eagle’s medium (DMEM) or Roswell Park Memorial Institute (RPMI) 1640 supplemented with 10% fetal bovine serum (FBS) and 1% antibiotics (100 U/mL penicillin and 100 μg/mL streptomycin) at 37 °C and exposed to an atmosphere of 5% CO_2_.

### CCA tissue collection

We collected 60 CCA samples legally from patients who underwent routine surgical procedures between 2010 and 2016 at The First Affiliated Hospital of Harbin Medical University. The histopathological diagnosis was based on the World Health Organization criteria. Clinical tumor typing was assigned based on the sixth edition of the TNM classification system published by the International Union Against Cancer. The histological grade of tumor differentiation was confirmed in accordance with the classification proposed by Edmondson and Steiner. Ethical approval was obtained from The First Affiliated Hospital of Harbin Medical University Research Ethics Committee and written informed consent was obtained from each patient. The detailed clinicopathological characteristics of the 60 CCA specimens used in this study are listed in Table 1.

**Table 1:** Correlation between TSPAN1 staining and clinicopathologic characteristics in 60 CCA patients

Variables	TSPAN1 staining		
	Positive(*n* = 42)	Negative/low(*n* = 18)	*P* value
	n(%)	n(%)	
Age (years)			
> 60	27(64.29%)	11(61.11%)	0.8151
≤ 60	15(35.71%)	7(38.89%)	
Gender			
Male	23(54.76%)	9(50.00%)	0.7347
Female	19(45.24%)	9(50.00%)	
Histological differentiation			
Well	10(23.81%)	8(44.44%)	0.2752
Moderate	15(35.71%)	5(27.78%)	
Poor	17(40.48%)	5(27.78%)	
TNM stage			
I-II	16(38.10%)	14(77.78%)	**0.0048**
III-IV	26(61.90%)	4(22.22%)	
CA19–9 (U/ml)			
≤ 37	13(30.95%)	5(27.78%)	0.8058
> 37	29(69.05%)	13(72.22%)	
Lymph node metastasis			
No	19(45.24%)	14(77.78%)	**0.0202**
Yes	23(54.76%)	4(22.22%)	
Distant metastasis			
No	29(69.05%)	17(94.44%)	**0.0331**
Yes	13(30.95%)	1(5.56%)	

Boldface indicates statistically significant values (*P* < 0.05)

### Lentivirus, antibodies, and reagents

The lentiviral vectors overexpressing the human *TSPAN1*, *miR-194-5p*, and *Snail* gene (LV-TSPAN1, LV-miR-194-5p, and LV-Snail, respectively) and their corresponding knockdown forms (LV-shTSPAN1, LV-anti-miR-194-5p, and LV-shSnail, respectively) were constructed and synthesized by Shanghai GeneChem Corporation (Shanghai, China). Empty vectors were used as the corresponding controls. The lentiviral vector encoding the human firefly luciferase gene was constructed and purchased from RiboBio Corporation (Guangzhou, China). The target sequences are listed in Additional file 1: Table S1. Information on the primary antibodies for the western blot, immunohistochemistry (IHC), immunofluorescence (IF), and co-immunoprecipitation (co-IP) analyses is provided in Additional file 1: Table S2. Detailed information of the primers and probes used in the quantitative reverse transcription-polymerase chain reaction (qRT-PCR) is listed in Additional file 1: Table S3. Laminin 5 and LY294002 were purchased from Abcam Corporation (Cambridge, Cambridgeshire, UK).

### Cell proliferation analysis and colony formation assay

Transfected cells were seeded in 96-well plates (1–2 × 10^3^ cells per well) and incubated overnight at 37 °C with 5% CO_2_ to allow attachment. Cell viability at various time points was measured using Cell Counting Kit-8 (CCK-8) assays (CK04–01; Dojindo Molecular Technologies, Inc., Japan). The primary medium in the wells was replaced with 100 μL of complete medium supplemented with 10 μL of CCK-8 reagent, and after incubating at 37 °C under 5% CO_2_ for 2 h, 450-nm absorbance was measured. The experiments were performed in triplicate. For the colony formation assay, transfected cells were seeded in six-well plates at a density of 500–800 cells/well and incubated for 14 days at 37 °C under an atmosphere of 5% CO_2_. Subsequently, the cells were fixed with 4% (*w*/*v*) paraformaldehyde, stained with 0.5% crystal violet, and counted.

### Wound healing assay

Cells were seeded in a six-well plate and cultured at 37 °C under an atmosphere of 5% CO_2_ until the cells had reached confluence. Cells were washed three times with phosphate-buffered saline (PBS), and the bottom of each well was scratched with a 200 μL pipette tip. An Eclipse TS100 microscope (Nikon, Tokyo, Japan) was used to capture the images at 0 and 24 h.

### Migration and invasion assays

A BD Falcon 24-well insert system (BD Biosciences, San Jose, CA, USA) was used to perform experiments. For the cell migration assay, 2–4 × 10^4^ cells were suspended in 500 μL serum-free medium, seeded in the upper chambers of the transwell, and then the culture medium containing 10% FBS was added to the lower chambers. For the Matrigel invasion assay, filters were precoated with 30 μL Matrigel (BD Biosciences, San Jose, CA, USA) for 3 h and the follow-up procedures were same as those for the migration assay. After incubation at 37 °C under an atmosphere of 5% CO_2_ for 24–48 h, the non-migrating or non-invading cells remained on the upper surface of the filters. Cells that penetrated the membrane filters were fixed in methanol, stained with crystal violet, and counted under a light microscope.

### Western blot analysis

Whole cell or tissue extracts were prepared using radioimmunoprecipitation assay (RIPA) buffer containing protease and phosphatase inhibitors. After determining the protein concentration, the samples were denatured, separated using sodium dodecyl-polyacrylamide gel electrophoresis, and then transferred onto polyvinylidene fluoride (PVDF) membranes (Invitrogen, Carlsbad, CA, USA). The membranes were probed with the antibodies listed in Additional file 1: Table S2. Protein bands were visualized using an enhanced chemiluminescence assay kit (Pierce, Rockford, IL, USA).

### QRT-PCR

Total RNA was extracted from cultured cells and clinical tissues using an RNeasy Mini kit (Qiagen, Valencia, CA, USA) according to the manufacturer’s protocol. Furthermore, complementary DNA was generated using the High-Capacity RT kit (Applied Biosystems, Foster City, CA, USA) after RNA quantification. The qRT-PCR assays were performed using Power SYBR Green PCR Master Mix (Life Technologies, Carlsbad, CA, USA), using an ABI Prism 7900HT instrument (Applied Biosystems, Carlsbad, CA, USA) with the primers listed in Additional file 1: Table S3 and normalized to the relative glyceraldehyde 3-phosphate dehydrogenase (GAPDH) mRNA level. For the miRNA analyses, a TaqMan® MicroRNA RT kit and TaqMan® Universal Master Mix II no UNG (Applied Biosystems, Carlsbad, CA, USA) were used to perform the RT and PCR, respectively. The miRNA expression levels were normalized to that of U6.

### IHC assay

The formalin-fixed and paraffin-embedded tissue sections were deparaffinized in xylene and rehydrated with a gradient ethanol series. Then, antigen retrieval was performed using an antigen-unmasking solution (citrate-based). Tissue sections were blocked and incubated with primary antibodies at optimal concentrations overnight at 4 °C. Then, the biotinylated sections were incubated with the secondary antibody (Vector Laboratories, Burlingame, CA, USA) for 1 h at room temperature. Lastly, the sections were stained with diaminobenzidine (DAB) kit (Vector Laboratories) and counterstained with hematoxylin (Sigma-Aldrich, St. Louis, MO, USA). The results were scored based on the intensity and the extent of staining. TSPAN1 staining intensity was scored as 0 (negative), 1 (weak), 2 (moderate) and 3 (strong). The staining extent was scored based on the percentage of positive cells using the following scale: 0 (negative), 1 (0.01–25%), 2 (25.01–50%), 3 (50.01–75%), and 4 (75.01–100%). The histologic score (H score) for each section was calculated with the following formula: histologic score = proportion score × intensity score. Thus, the total score could be 0, 1, 2, 3, 4, 6, 8, 9, or 12, and the staining could be classified as negative/low (0, 1, 2, 3, 4) or positive (6, 8, 9, 12).

### IF assay

For the IF assay, transfected cells were fixed with 4% (*w*/*v*) paraformaldehyde for 10 min, permeabilized with 0.1% (*v*/*v*) Triton X-100 for 20 min at ambient temperature, and blocked in 10% normal goat serum for 30 min. Cells were then incubated with primary antibodies overnight at 4 °C. After thorough washing with PBS, the cells were incubated with fluorescent secondary antibodies (Invitrogen, Eugene, OR, USA) for 1 h at room temperature. Finally, the cells were washed and counterstained with 4′,6-diamidino-2-phenylindole (Vector Laboratories, Burlingame, CA, USA) and then images were captured using a DMRA fluorescence microscope.

### Co-IP assay

Cells were seeded in six-well plates, incubated with 200–400 μL ice-cold IP lysis buffer per well on ice for 5 min with periodic mixing, and supplemented with 10 μL/mL Halt™ protease and phosphatase inhibitor cocktail (Thermo Scientific, Waltham, MA, USA). Then, we transferred the lysate to a microcentrifuge tube, which was centrifuged at 13000×g for 10 min to pellet the cell debris at 4 °C. A Dynabeads™ protein G IP kit and Magnet Starter Pack (Thermo Scientific) were used to immunoprecipitate the Dynabeads®-Ab-Ag complex following the manufacturer’s protocol, and samples were analyzed using western blotting as previously described.

### Luciferase assay

In brief, a luciferase assay kit (Promega, Madison, WI, USA) was used to evaluate the luciferase activity. Cells were seeded in 24-well plates and incubated for 24 h. For the transfection, specific plasmids were cotransfected with 1 ng pRL-TK Renilla luciferase plasmid into the cells. Lastly, the Dual-Luciferase Reporter Assay system (Promega) used to measure the luciferase activity after 48 h according to the manufacturer’s instructions.

### Animal studies

Male BALB/c athymic nude mice (4–6-week-old) were obtained from the Experimental Animal Center of Shanghai Institute for Biological Sciences and maintained in a pathogen-free environment according to the institutional guidelines for animal care. To establish a subcutaneous xenograft model, 5 × 10^6^ cells suspended in 150 μL PBS were subcutaneously injected into the flank of mice. The tumors were excised after 6 weeks to calculate their volumes.

For the in vivo metastasis assays, intrahepatic, peritoneal, and pulmonary metastases were established. The intrahepatic metastasis assay was performed by injecting 5 × 10^6^ cells in 200 μL PBS into the mouse livers through the spleen parenchyma. To avoid intrasplenic tumor growth, the mouse spleens were removed. After 4 weeks, the mice were euthanized, and their livers were harvested to count the nodules.

To evaluate the peritoneal metastasis, 5 × 10^6^ CCA cells in 200 μL PBS were inoculated into the intraperitoneal cavity of the mice, which were subsequently euthanized, dissected, and representative images were captured using a camera, 4 weeks after the injections.

Furthermore, 5 × 10^6^ cells were injected into nude mice through the tail vein, and 6 weeks later, tumor formation and metastasis in the lungs were measured using a Berthold NIGHTOWL LB983 imaging machine with D-luciferin (Xenogen, Hopkinton, MA, USA). After imaging, all the mice were euthanized, the lungs were excised, and the metastatic nodules were counted following hematoxylin and eosin staining (H&E) staining.

### Statistical analysis

The statistical analysis was performed using the statistical package for the social sciences (SPSS) 16.0 software (SPSS, Chicago, IL, USA) and the GraphPad Prism software package (v. 6.01, San Diego, CA, USA). The results are presented as means ± SD. The variance between the two groups was analyzed using Student’s *t*-tests, and *p* < 0.05 was considered statistically significant.

## Results

### TSPAN1 is frequently upregulated in human CCA

**Fig. 1:**
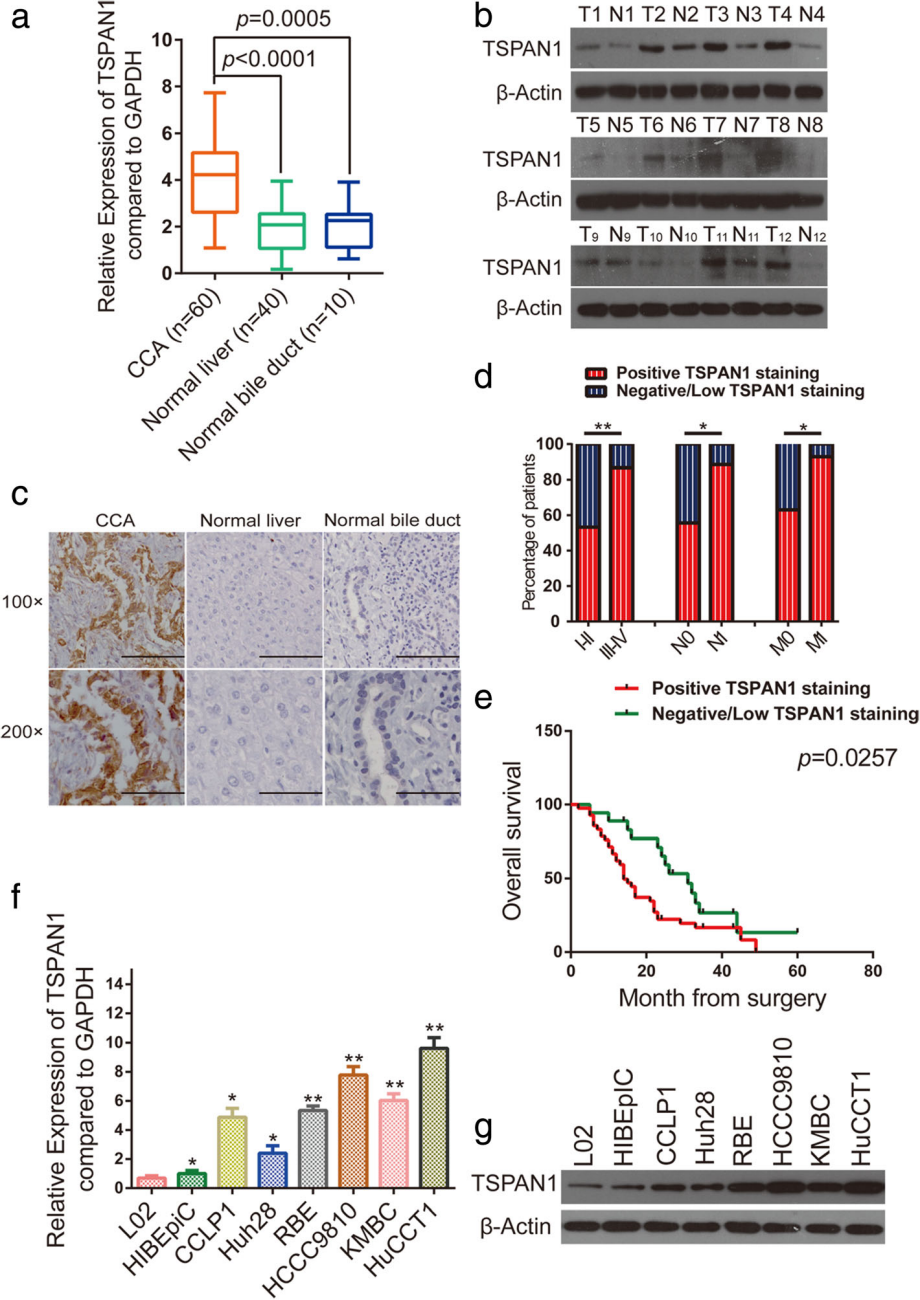
Tetraspanin 1 (TSPAN1) is frequently upregulated in human cholangiocarcinoma (CCA). (**a**) TSPAN1 mRNA level was analyzed in 60 CCA and paracancerous, 40 normal liver, and 10 normal bile duct tissue specimens using real-time quantitative reverse transcription-polymerase chain reaction (qRT-PCR). (**b** and **c**) Western blot and immunohistochemical (IHC) analyses of TSPAN1 protein expression in CCA tissues and adjacent normal tissues. Scale bars: 100× = 100 μm; 200× = 50 μm. (**d**) TSPAN1 positive staining was associated with poor clinicopathological features, including TNM stage (I–II and III–IV), lymph node status (N0 or N1), and metastasis status (M0 or M1). (**e**) A Kaplan-Meier analysis of overall survival (OS) in patients with different staining of TSPAN1. (**f** and **g**) Relative TSPAN1 levels in L02, HIBEpiC, and six CCA cells were analyzed using qRT-PCR and western blot. Data are means ± SD of three independent experiments. *p < 0.05, **p < 0.01. T: tumor; N: normal paracancerous tissue

QRT-PCR results showed that TSPAN1 mRNA was increased in human CCA tissues (*n* = 60) compared with that in the liver (*n* = 40, *p* < 0.0001) and bile duct samples (*n* = 10, *p* = 0.0005, Fig. 1a). Besides, TSPAN1 protein was expressed at a significantly higher level in CCA samples by western blot analysis (Fig. 1b). IHC analysis was also used to determine that TSPAN1 expression showed strong staining in CCA tissues with a total positive proportion of 70% (42/60), which was higher than the 13.3% (4/30) observed in the liver and 20% (6/30) in normal bile duct samples (Fig. 1c). Accordingly, we divided the patients with CCA into two groups (Additional file 2: Table S4): the TSPAN1-positive and -negative/low groups. There were statistical associations between TSPAN1 and clinicopathological characteristics (Table 1). The statistical analyses revealed that TSPAN1 expression was positively correlated with the advanced disease stage (*p* = 0.0048), lymph node status (*p* = 0.0202), and metastasis (*p* = 0.0331, Fig. 1d). A Kaplan-Meier curve showed that patients in TSPAN1 positive staining group exhibited a decreased trend in overall survival (OS) than TSPAN1 negative/low group (*p* = 0.0257, Fig. 1e). To strengthen the independent prognostic significance of TSPAN1, we analyzed the OS using a Cox regression analysis. The multivariate analysis revealed TSPAN1 as an independent prognostic factor for overall survival in CCA (Additional file 3: Figure S1).

In addition, TSPAN1 protein (Fig. 1f) and mRNA levels (Fig. 1g) were observed to increase gradually in CCA cells with high metastatic potential (KMBC, HCCC9810, and HuCCT1) to CCA cells with low metastatic potential (CCLP1, RBE, and Huh28) and, ultimately, to normal liver (L02) and normal human intrahepatic biliary (HIBEpiC) cell lines [21]. These results indicated that TSPAN1 expression was frequently upregulated in human CCA.

### TSPAN1 promotes CCA cell proliferation and tumorigenicity in vitro and in vivo

**Fig. 2:**
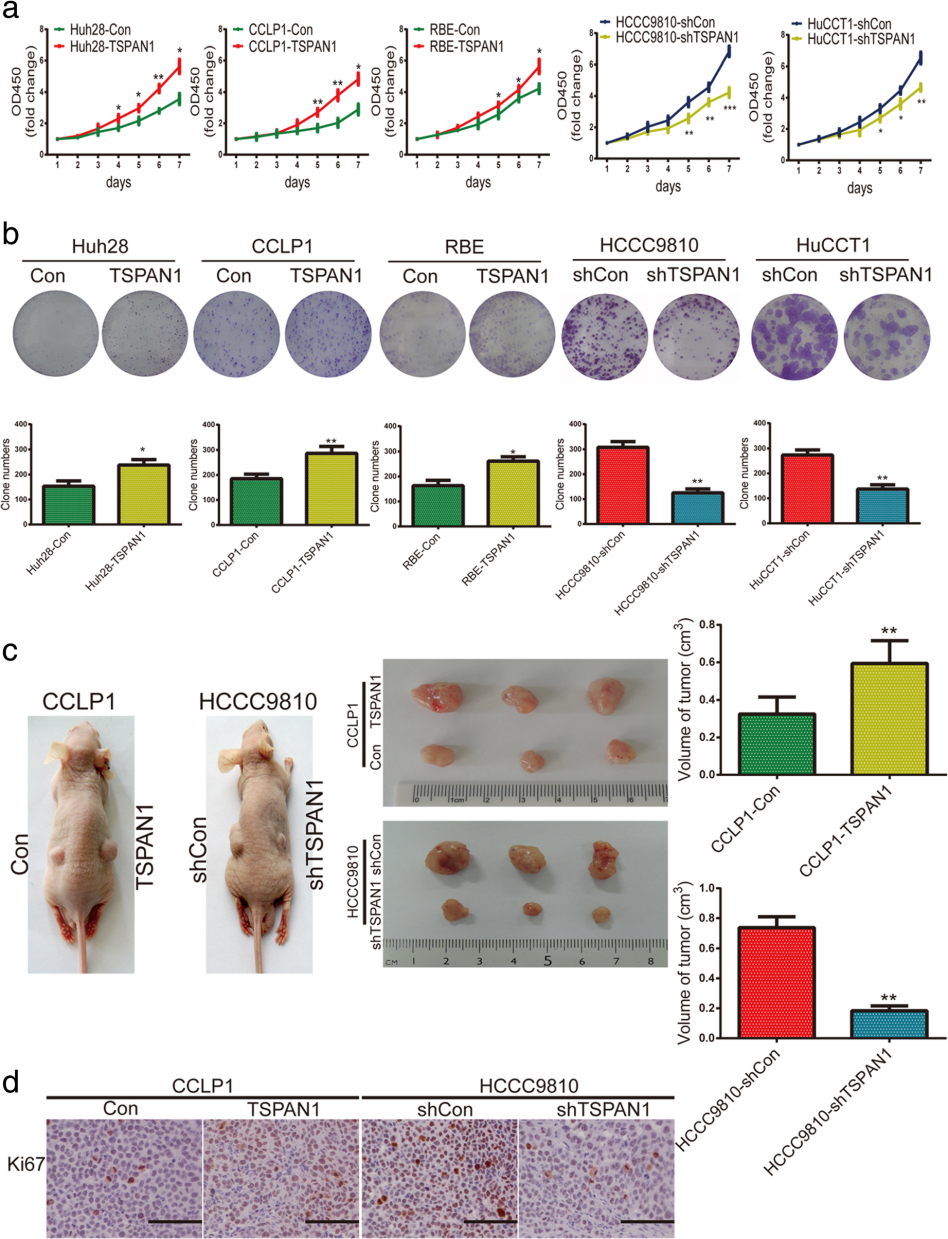
Tetraspanin 1 (TSPAN1) promotes CCA cell proliferation and tumorigenicity in vitro and in vivo. (**a**) Proliferation rate was analyzed by CCK-8 assay of indicated CCA cells (Con, LV-TSPAN1, shCon, and LV-shTSPAN1). (**b**) Representative images of colony formation assays are shown in the upper panels; the number of foci was counted as shown in the panels below. (**c**) TSPAN1 upregulation enhanced CCLP1 cell xenograft tumor growth in nude mice while TSPAN1 knockdown reduced HCCC9810 growth. (**d**) Immunohistochemical (IHC) staining was used to detect Ki-67 expression in xenograft tissues (200× magnification), scale bars = 50 μm. Data are means ± SD of three independent experiments. *p < 0.05 and **p < 0.01

To investigate the role of TSPAN1 in CCA cell proliferation and tumorigenicity, TSPAN1 was overexpressed or knocked down using lentivirus transfection. After transfection, TSPAN1 was overexpressed in Huh28, CCLP1, and RBE cell lines, and in HCCC9810 and HuCCT1 cells, TSPAN1 expression was notably decreased by lenti-shTSPAN1–3 and, thus, shTSPAN1–3 was chosen for further experiments (Additional file 4: Figure S2a). Overexpressing TSPAN1 promoted Huh28, CCLP1, and RBE cell proliferation, while silencing had the opposite effect in HCCC9810 and HuCCT1 cells as measured using CCK8 proliferation analysis (Fig. 2a). Similar to its effects on cell proliferation, in a colony formation assay, compared with control group, TSPAN1 significantly increased colony number in Huh28, CCLP1, RBE, and TSPAN1 silencing resulted in fewer colonies in HCCC9810 and HuCCT1 cells (Fig. 2b).

Furthermore, a subcutaneous xenograft mouse model was established to observe the role of TSPAN1 in tumorigenesis in vivo (*n* = 5/group). The processed and corresponding CCA control group cells were injected into the flanks of nude mice on different sides respectively. After 6 weeks, tumor volume was increased in the CCLP1-TSPAN1 group compared with that in the control group and was decreased in mice inoculated with HCCC9810-shTSPAN1 cells (Fig. 2c). An IHC assay was performed to evaluate the Ki-67 expression, which was upregulated in the CCLP1-TSPAN1 group and downregulated in the HCCC9810-shTSPAN1 group relative to their respective controls (Fig. 2d and Additional file 4: Figure S2b). These results revealed that TSPAN1 accelerated CCA cell proliferation and tumorigenesis in vitro and in vivo.

### TSPAN1 promotes CCA cell migration and invasion in vitro and in vivo

**Fig. 3:**
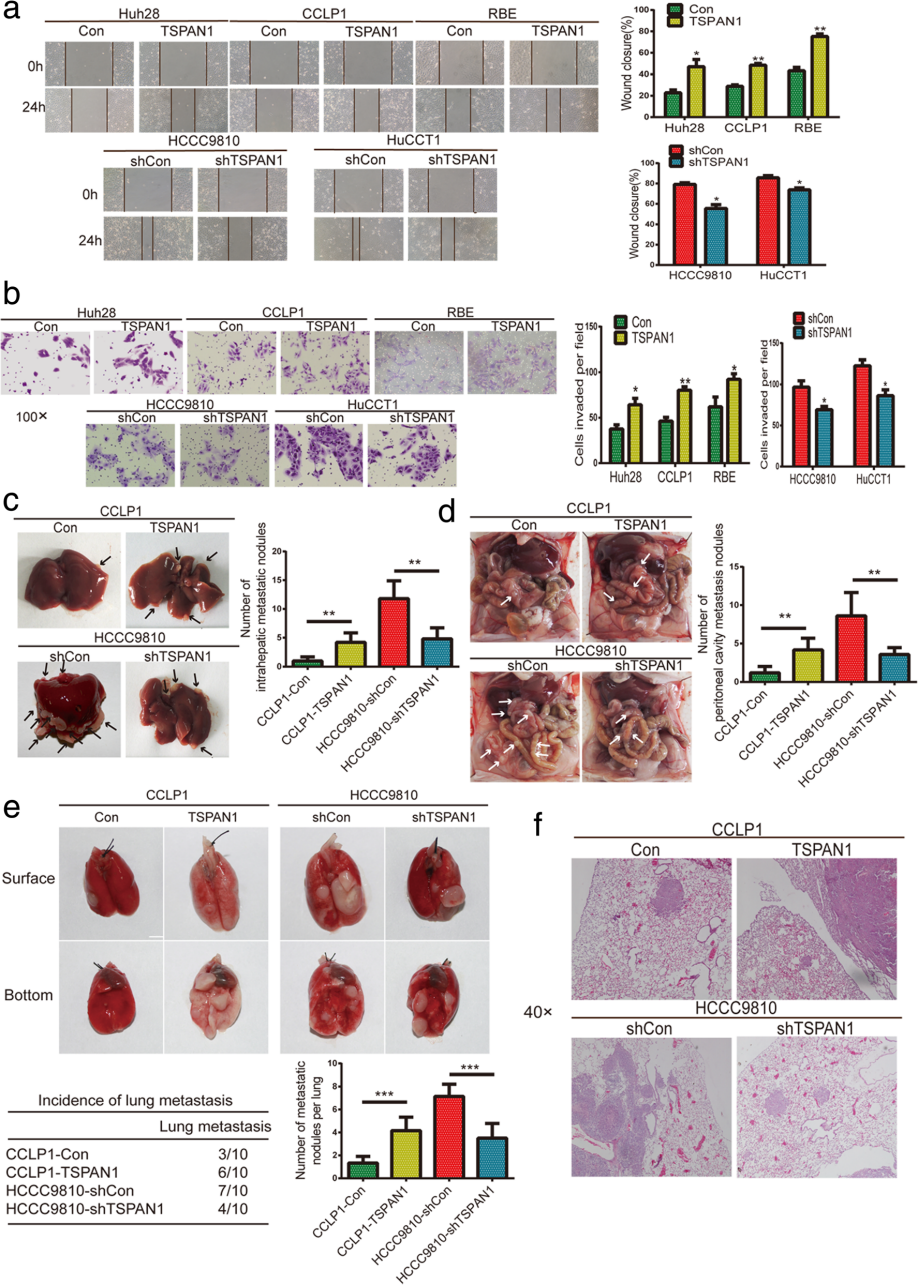
Tetraspanin 1 (TSPAN1) promotes cholangiocarcinoma (CCA) cell migration and invasion in vitro and in vivo. (**a**) Overexpressing TSPAN1 promoted CCA cells migration, whereas silencing TSPAN1 suppressed migration in wound healing assay; representative images were captured at 0 and 24 h. (**b**) Representative images of invasion assays (100× magnification) for CCA cell lines. (**c**) Images of intrahepatic metastatic nodules originating from CCLP1 and HCCC9810 cells and number of nodules are shown in the left panels. (**d**) Images of peritoneal cavity metastasis of CCLP1 and HCCC9810 cells and number of nodules are summarized in the right panels. (**e**) Pulmonary metastatic nodules are shown in the upper panels, and number and incidence of nodules are displayed in the lower panels. (**f**) Representative hematoxylin and eosin (H&E) images of lung metastases (40× magnification) are shown for indicated cell lines. Data are means ± SD of three independent experiments. *p < 0.05, **p < 0.01, and ***p < 0.001

A potential function of TSPAN1 was implied in tumor migration and invasion based on the above clinicopathological analysis. We selected low (Huh28, CCLP1, and RBE) and high (HCCC9810 and HuCCT1) metastatic cells for the experiments. The wound healing assay showed that TSPAN1 upregulation enhanced CCA cell migration relative to that of control cells, whereas TSPAN1 knockdown had the opposite effect (Fig. 3a). Moreover, Matrigel-coated (for invasion) or -uncoated (for migration) transwell assays revealed that TSPAN1 overexpression drastically increased the invasiveness and migration of low metastatic cells, whereas its knockdown dramatically decreased the invasiveness and migration of highly metastatic cells (Fig. 3b and Additional file 5: Figure S3).

To investigate the role of TSPAN1 in metastasis in vivo, liver metastasis (n = 5/group), peritoneal cavity metastasis (n = 5/group), and lung metastasis (*n* = 10/group) models were established. Liver metastasis was significantly increased in mice implanted with CCLP1-TSPAN1 cells compared to that of the control group, and the number of tumor nodules was dramatically decreased in mice implanted with HCCC9810-shTSPAN1 cells compared to that of shCon cells 4 weeks after operation (Fig. 3c). Results of peritoneal cavity metastasis also showed that TSPAN1 overexpression drastically enhanced the number of metastatic nodules and TSPAN1 knockdown dramatically reduced the number of metastatic nodules (Fig. 3d). We then injected stably transfected cell lines into nude mice via the tail vein and monitored the development of metastatic nodules in the lungs. After 6 weeks, the number of nodules and incident rate were drastically increased with CCLP1-TSPAN1 cells and were significantly decreased with HCCC9810-shTSPAN1 cells (Fig. 3e) compared to the control cells. The presence of pulmonary metastatic nodules was also tested using H&E of dissected lung tissue (Fig. 3f). These data provided evidence that TSPAN1 was involved in promoting CCA cell metastasis in vitro and in vivo.

### TSPAN1 induces EMT in CCA

**Fig. 4:**
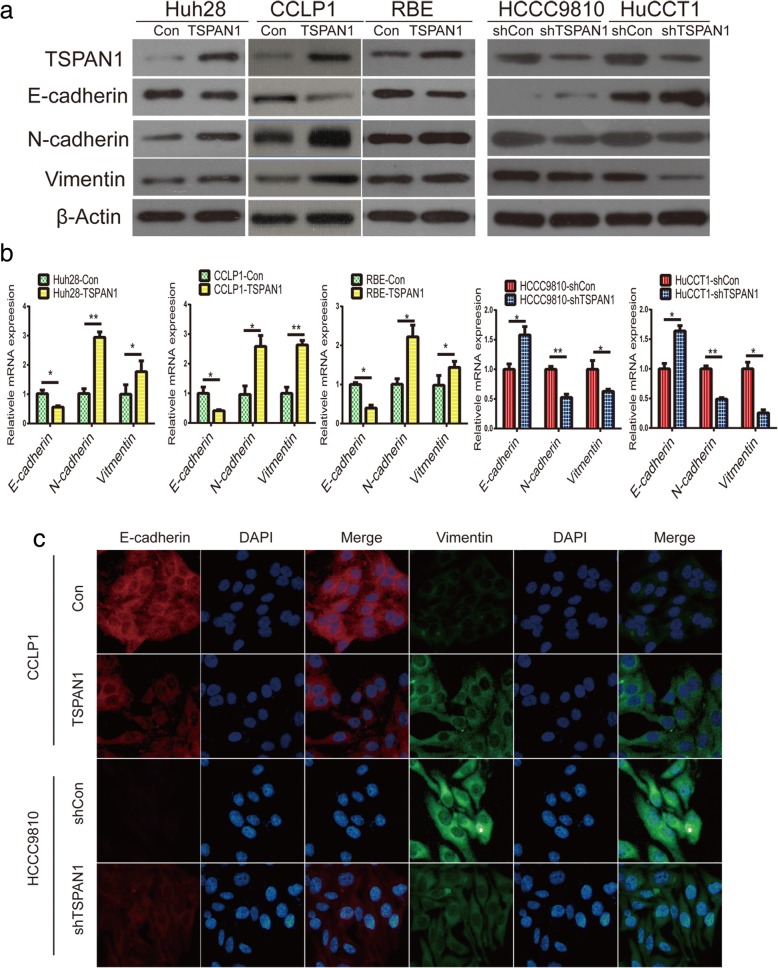
Tetraspanin 1 (TSPAN1) induces epithelial-mesenchymal transition (EMT) in cholangiocarcinoma (CCA). (**a** and **b**) Expression of E-cadherin, N-cadherin, and vimentin was evaluated using western blot and real-time quantitative reverse transcription-polymerase chain reaction (qRT-PCR) in indicated processed cells and their corresponding control group. (**c**) E-cadherin and vimentin protein expression and subcellular localization were determined using immunofluorescence (IF) in CCLP1 and HCCC9810 cells. Data are means ± SD of three independent experiments. *p < 0.05 and **p < 0.01

EMT is a reversible dynamic process during which epithelial cells gradually adopt structural and functional characteristics of mesenchymal cells. More specifically, EMT is an early event of metastasis that is required for tumor cell migration and invasion from the primary tumor. To investigate whether TSPAN1 controls the EMT process, the expression of epithelial and mesenchymal markers were examined. The results of the western blot analysis and qRT-PCR showed that the epithelial marker E-cadherin was downregulated, whereas the mesenchymal markers N-cadherin and vimentin were upregulated in LV-TSPAN1 cells compared to those in control cells (Fig. 4a and b). Conversely, TSPAN1 silencing increased epithelial marker expression while suppressing that of mesenchymal markers in LV-shTSPAN1 cells. Similar results were observed with IF analyses (Fig. 4c). In phase-contrast images, we discovered that CCLP1-TSPAN1 cells showed a spindle-like mesenchymal morphology, whereas HCCC9810-shTSPAN1 were round with a more epithelial cell-like morphology (Additional file 6: Figure S4). Therefore, these data indicated that TSPAN1 induced EMT process in CCA.

### TSPAN1 is a downstream target of miR-194-5p

**Fig. 5:**
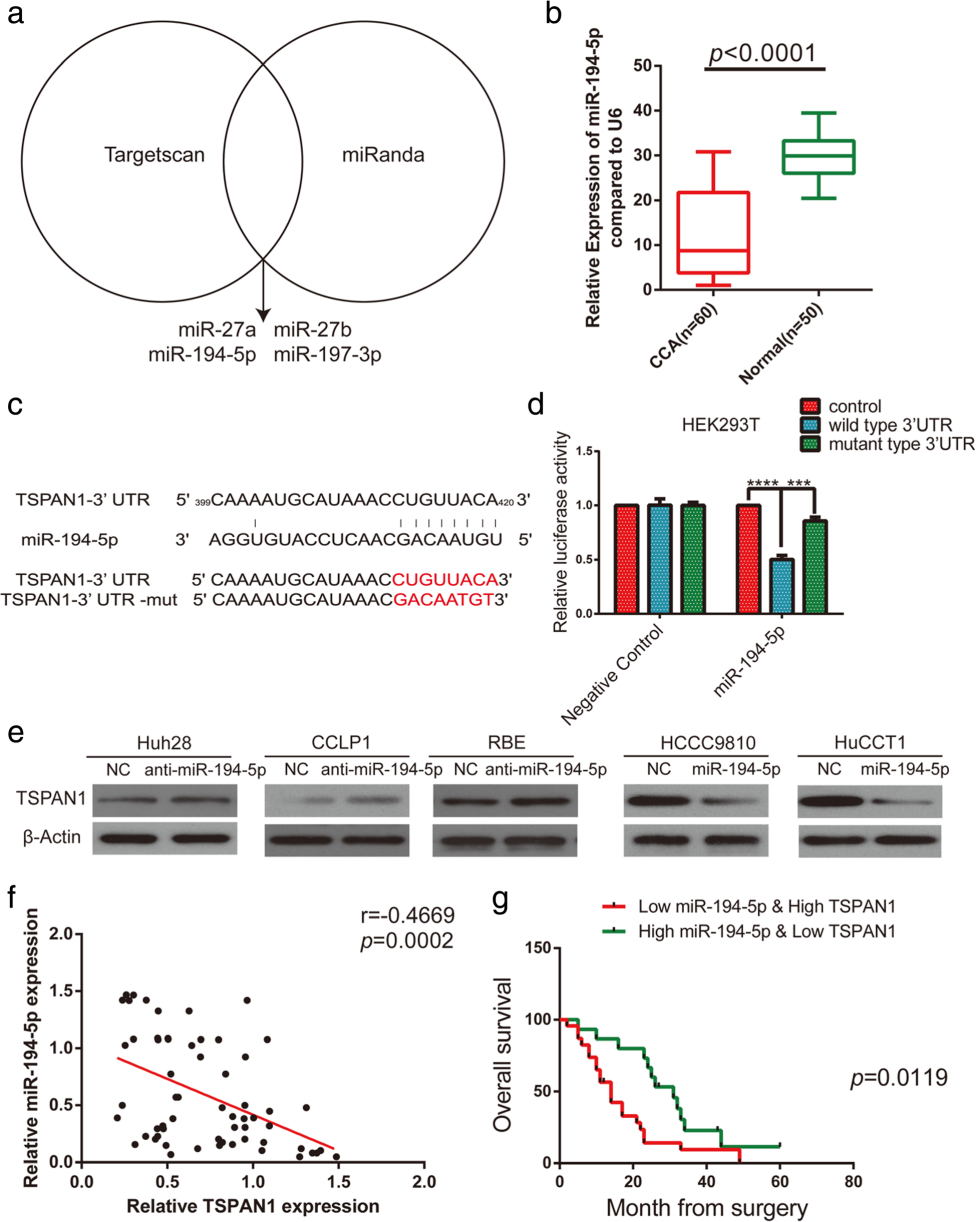
Tetraspanin 1 (TSPAN1) is a downstream target of micro RNA (miR)-194-5p. (**a**) Analysis using TargetScan and miRanda revealed several miRNAs might regulate TSPAN1. (**b**) Human cholangiocarcinoma (CCA) tissues displayed significantly lower miR-194-5p levels than adjacent nontumor tissues did. (**c**) Binding site of miR-194-5p in wild-type (WT) 3′-untranslated region (UTR) of TSPAN1 and corresponding mutant type were constructed. (**d**) Luciferase reporter assay showed luciferase activity of HEK293T transfected with WT 3′-UTR was obviously inhibited by miR-194-5p overexpression. (**e**) miR-194-5p overexpression or silencing decreased or increased TSPAN1 protein in indicated cells. (**f**) Correlation analysis indicated an inverse correlation between miR-194-5p and TSPAN1 expression. (**g**) Kaplan-Meier analysis indicates that combination of high TSPAN1 and low miR-194-5p predicts poorer overall survival rate than low TSPAN1 and high miR-194-5p. All data are means ± SD of three separate experiments. ***p < 0.001 and ****p < 0.0001

Next, we investigated the relationship between miRNA deregulation and TSPAN1 upregulation in human CCA. Several possible miRNAs as research targets were identified that might be upstream regulators of TSPAN1 using TargetScan and miRanda for the analysis (Fig. 5a). After qRT-PCR method, miR-194-5p was selected as the most frequently low expressed miRNA in CCA tissues (*p* < 0.0001) among other miRNAs including miR-27a, miR-27b, and miR-197-3p (Fig. 5b and Additional file 7: Figure S5a). In cell lines, a lower miR-194-5p level was detected in CCA cells than that in the HIBEpiC and L02 cells (Additional file 7: Figure S5b). Moreover, we designed wild-type (WT) and mutated TSPAN1 3′-untranslated region (UTR)-coupled luciferase reporters to verify the binding between miR-194-5p and TSPAN1 3′-UTR (Fig. 5c). In HEK293T cells, the luciferase activity was obviously suppressed by miR-194-5p in the WT 3′-UTR group, but no significant change was observed in the mutant 3′-UTR group (Fig. 5d), indicating that TSPAN1 is a direct target of miR-194-5p. After the lentivirus transfection and qRT-PCR, Huh28-anti-miR-194-5p, CCLP1-anti-miR-194-5p, RBE-anti-miR-194-5p and HCCC9810-miR-194-5p, HuCCT1-miR-194-5p complexes were constructed with their corresponding control groups (Additional file 7: Figure S5c). Subsequently, the results were corroborated using western blot analysis, which showed that miR-194-5p weakened the expression of TSPAN1 protein (Fig. 5e). The statistical analyses revealed that low miR-194-5p expression was positively correlated with the advanced disease stage (*p* = 0.0073), lymph node status (*p* = 0.0044, Table 2). At last, the Pearson correlation analysis demonstrated a negative correlation between TSPAN1 and miR-194-5p expression in CCA clinical samples (*r* = − 0.4669, *p* = 0.0002, Fig. 5f) and the combination of high TSPAN1 level and low miR-194-5p level predicted poor prognosis in patients (*p* = 0.0119, Fig. 5g). Therefore, our tests suggested that TSPAN1 was negatively regulated by miR-194-5p in CCA.

**Table 2:** Correlation between miR-194-5p expression and clinicopathologic chacteristics in 60 CCA patients

Variabls	miR-194-5p expression		
	Low(*n* = 45)	High(*n* = 15)	*P* value
	n(%)	n(%)	
Age (years)			
> 60	28(62.22%)	10(66.67%)	0.7571
≤ 60	17(37.78%)	5(33.33%)	
Gender			
Male	25(55.56%)	7(46.67%)	0.5501
Female	20(44.44%)	8(53.33%)	
Histological differentiation			
Well	12(26.67%)	6(40.00%)	0.2940
Moderate	14(31.11%)	6(40.00%)	
Poor	19(42.22%)	3(20.00%)	
TNM stage			
I-II	18(40.00%)	12(80.00%)	**0.0073**
III-IV	27(60.00%)	3(20.00%)	
CA19–9 (U/ml)			
≤ 37	11(24.44%)	7(46.67%)	0.1038
> 37	34(75.56%)	8(53.33%)	
Lymph node metastasis			
No	20(44.44%)	13(86.67%)	**0.0044**
Yes	25(55.56%)	2(13.33%)	
Distant metastasis			
No	32(71.11%)	14(93.33%)	0.0780
Yes	13(28.89%)	1(6.67%)	

Boldface indicates statistically significant values (*P* < 0.05)

### MiR-194-5p inhibits CCA metastasis and EMT

**Fig. 6:**
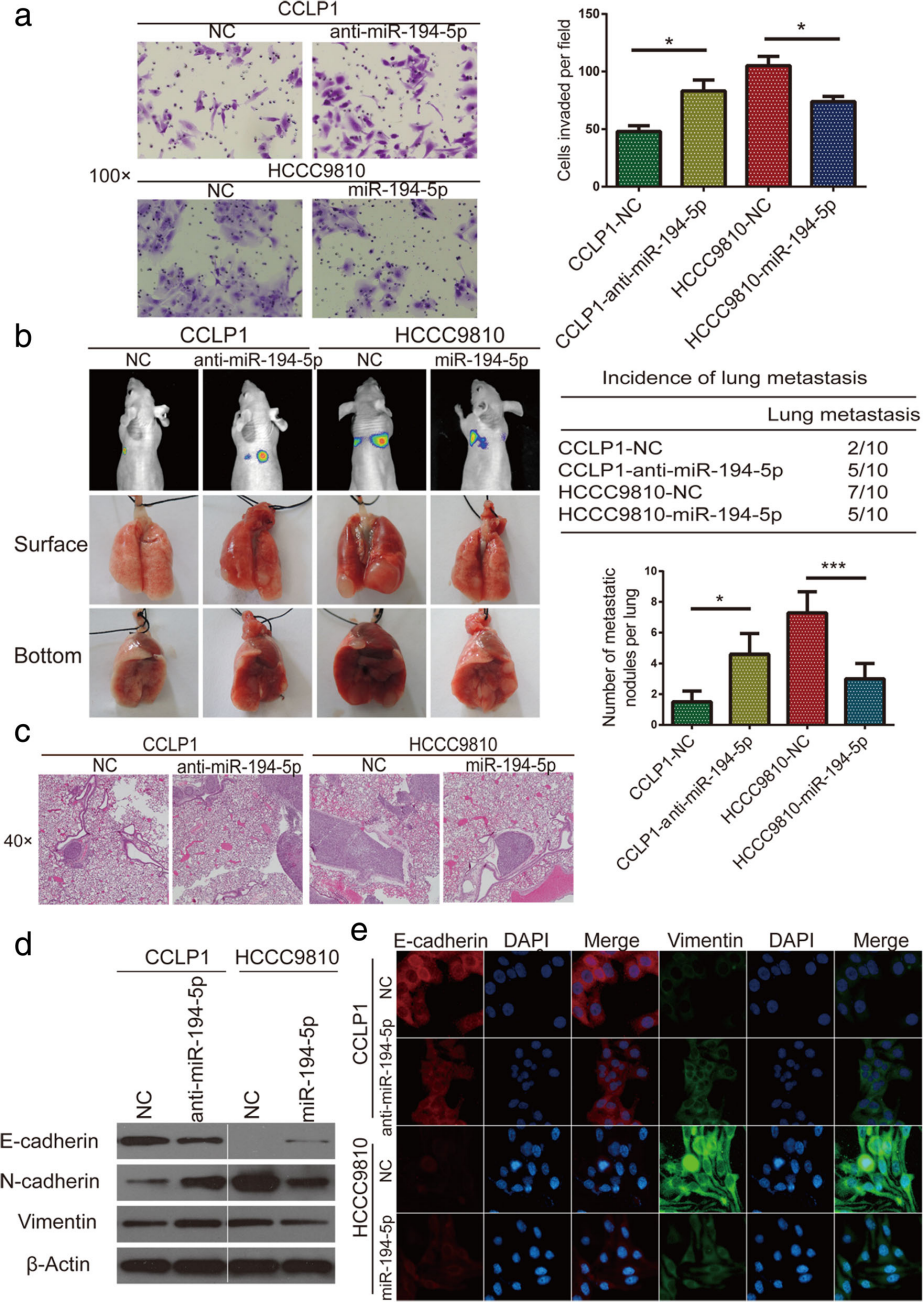
MicroRNA (miR)-194-5p inhibits cholangiocarcinoma (CCA) metastasis and epithelial-to-mesenchymal transition (EMT). (**a**) Matrigel-coated transwell assay (100× magnification) showed miR-194-5p inhibited cell invasion in CCLP1 and HCCC9810 lines. (**b** and **c**) Results indicate miR-194-5p suppressed lung metastasis in vivo, as determined using hematoxylin and eosin (H&E) staining (40× magnification). (**d** and **e**) Expression of epithelial-mesenchymal transition (EMT)-related markers was assessed using western blot and immunofluorescence (IF) analyses. Experiments were performed three times and data are means ± SD. *p < 0.05 and ***p < 0.001

To examine the function of miR-194-5p in CCA, wound healing and transwell assays as well as in vivo pulmonary metastasis (*n* = 10/group) analysis were performed with stably transfected cell lines (CCLP1-anti-miR-194-5p, CCLP1-NC, HCCC9810-miR-194-5p, and HCCC9810-NC). In wound healing assay, compared with control group, knockdown of miR-194-5p increased the motility of CCLP1, on the contrary, miR-194-5p overexpression significantly reduced migrated distances of HCCC9810 (Additional file 8: Figure S6a). As examined both in transwell migration and Matrigel invasion assays, knockdown of miR-194-5p promoted the migration and invasion of CCLP1 cells, which remarkably increased the number of invasive or migratory cells compared to the control group. In contrast, overexpression of miR-194-5p in HCCC9810 cell decreased their migratory and invasive capability (Fig. 6a and Additional file 8: Figure S6b). In lung metastasis and H&E staining experiment, more and bigger lung metastases nodules were observed in mouse model constructed using CCLP1-anti-miR-194-5p cells than those using CCLP1-NC cells, in contrast, less and smaller lung metastases nodules were observed in mouse model constructed using HCCC9810-miR-194-5p cells than those using HCCC9810-NC cells (Fig. 6b and c). These results showed that the effect of miR-194-5p on metastasis was contrary to that of TSPAN1 in CCA. Then we determined E-cadherin, N-cadherin, and vimentin content using western blot (Fig. 6e) and IF (Fig. 6f) analysis, showing silencing miR-194-5p in CCLP1 suppressed the expression of E-cadherin and increased N-cadherin and vimentin, and miR-194-5p overexpression in HCCC9810 cell had opposite effect. These results suggested that miR-194-5p inhibited EMT-related metastasis in CCA cells.

### TSPAN1 interacts with integrin α6β1

**Fig. 7:**
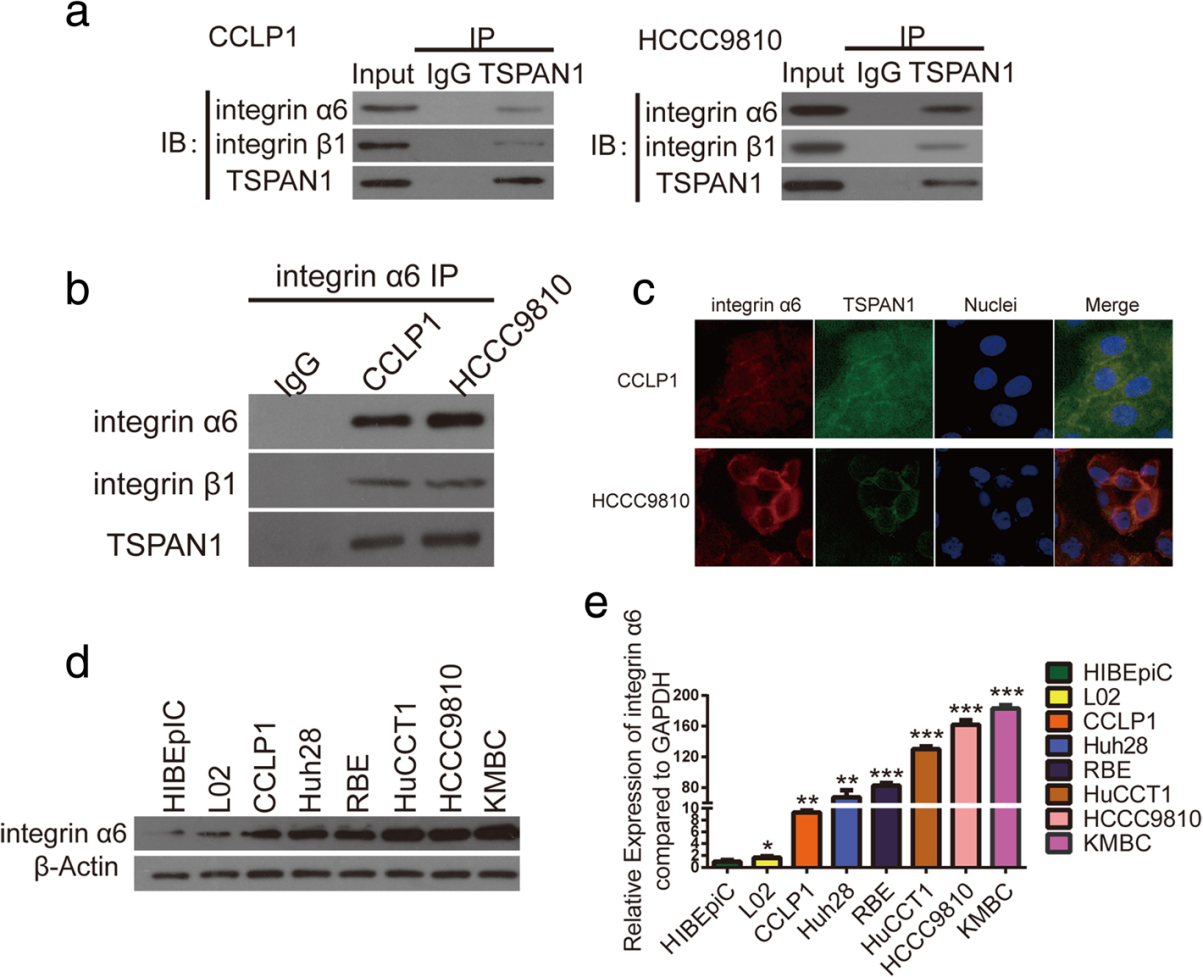
Tetraspanin 1 (TSPAN1) interacts with Integrin α6β1. (**a** and **b**) Co-immunoprecipitation (IP) showed TSPAN1 complexed with integrin α6β1. (**c**) Co-localization of TSPAN1 and integrin α6 was confirmed using immunofluorescence (IF) staining in CCLP1 and HCCC9810 cells. (**d** and **e**) Western blot and real-time quantitative reverse transcription-polymerase chain reaction (qRT-PCR) experiments showed that integrin α6 expression was higher in CCA cells than in the normal cells. Experiments were performed three times and data are means ± SD. *p < 0.05, **p < 0.01, and ***p < 0.001

A unique feature of TSPANs is that their functionality requires the formation of a complex with various integrins, and they are essential for integrin compartmentalization, internalization, recycling, and signaling. Therefore, we selected several integrins previously reported to function as oncoproteins in CCA, including α2β1, α5β1, α6β1, α6β4, and αVβ6, which TSPAN1 was likely to interact with [10, 22–24]. Co-IP assays showed that TSPAN1 and integrin α6β1 interacted with each other in CCA cells among these integrins (Fig. 7a, b and Additional file 9: Figure S7) and IF assays exhibited TSPAN1 and integrin α6 were co-expressed mainly on CCA cell membrane (Fig. 7c). To evaluate integrin α6 content in CCA and normal cells, western blot (Fig. 7d) and qRT-PCR (Fig. 7e) analyses were performed to show that integrin α6 expression was also higher in CCA cells than in the normal cells. This evidence indicated that TSPAN1 interacted with integrin α6β1 and might promote CCA progression by modulating integrin α6β1 downstream signaling.

### TSPAN1 enhances PI3K/AKT/GSK-3β/snail/PTEN feedback loop

**Fig. 8:**
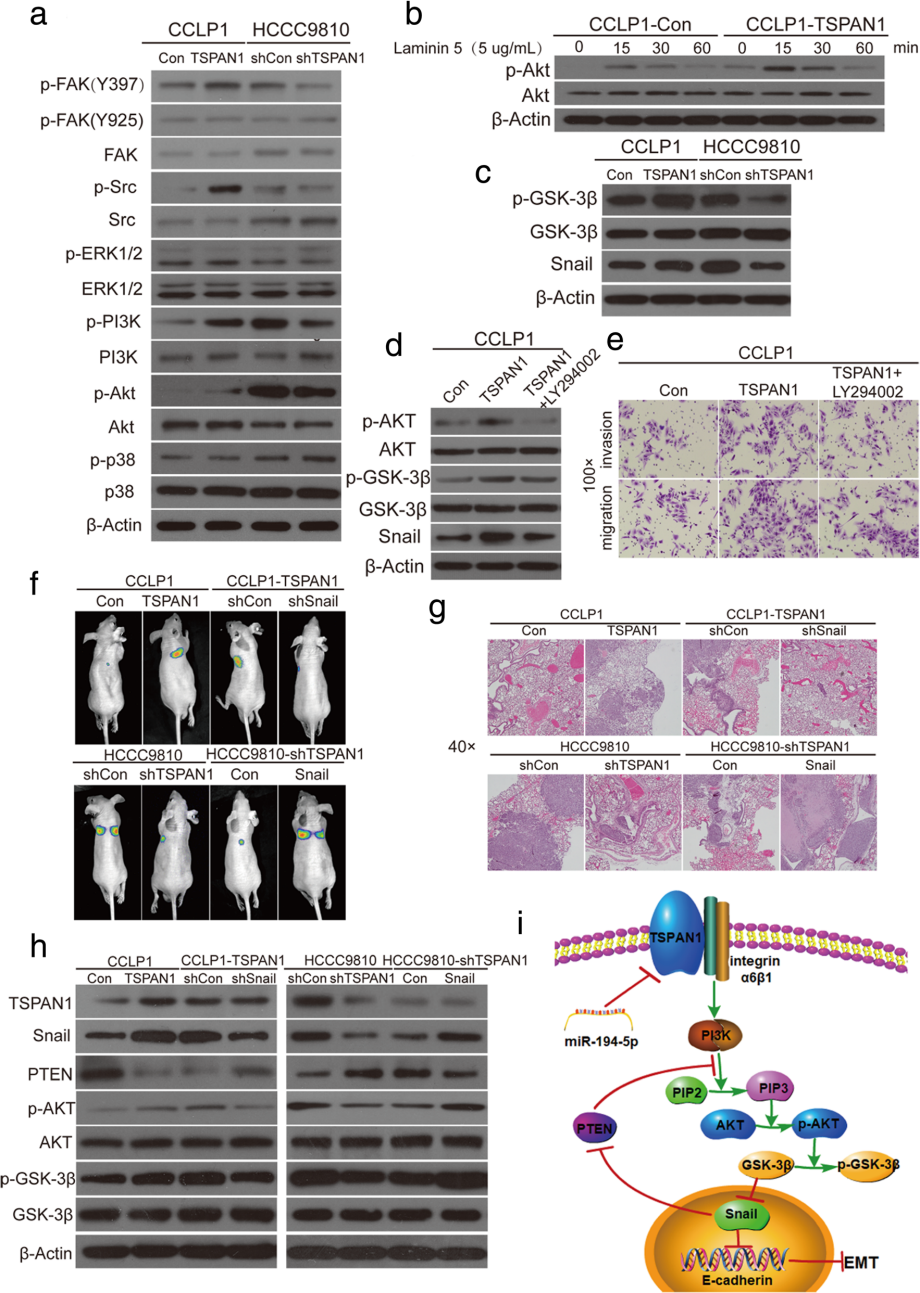
Tetraspanin 1 (TSPAN1) enhances phosphoinositide-3-kinase (PI3K)/AKT/glycogen synthase kinase (GSK)-3β/snail family transcriptional repressor (Snail)/phosphatase and tensin homolog feedback loop. (**a**) FAK, Src, extracellular signal-regulated kinase 1 and 2 (ERK1/2), PI3K, AKT and p38 and their phosphorylation were assayed in modified CCLP1 and HCCC9810 and their control cells. (**b**) CCLP1-Con and CCLP1-TSPAN1 cells were treated with laminin 5 (5 μg/ml) for 0, 15, 30, 60 min, and time-response experiment showed significant difference in p-AKT. (**c**) Phosphorylation of GSK-3β and Snail were examined in indicated cells. (**d**) Western blot analysis demonstrated that the PI3K inhibitor LY294002 effectively decreased expression levels of p-AKT, p-GSK-3β, and Snail induced by TSPAN1. (**e**) Stable CCLP1-TSPAN1 cells were treated with LY294002 and subjected to migration and invasion assays (100× magnification). (**f**) In vivo lung metastatic assay. Nude mice were injected with eight luciferase-expressing cell lines (CCLP1-TSPAN1 and CCLP1-TSPAN1 plus LV-shSnail, HCCC9810-shTSPAN1 and HCCC9810-shTSPAN1 plus LV-Snail, and their respective control cells) via the tail vein. Tumors were monitored using bioluminescence imaging. (**g**) Histological analyses of lung metastatic tumors by hematoxylin and eosin (H&E) staining. Images showing representative H&E staining of lung tissue samples from the different experimental groups. (**h**) Western blot analyses showed PTEN expression altered inversely with Snail change in CCLP1 and HCCC9810 cells. Knockdown of Snail expression using LV-shSnail in CCLP1-TSPAN1 cells significantly enhanced PTEN expression and attenuated the AKT/GSK-3β pathway induced by TSPAN1. In contrast, upregulation of Snail using LV-Snail markedly decreased PTEN and enhanced the pathway in HCCC9810-shTSPAN1 cells. (**i**) Schematic presentation of mechanism underlying TSPAN1-mediated CCA metastasis. Experiments were performed three times and data are means ± SD

The key component of canonical integrin signaling cascade is focal adhesion kinase (FAK). When integrins bind to their specific ligands, they recruit FAK by their β subunit, leading to FAK autophosphorylation and its association with Src. This process activates both kinases and further activates several intracellular signaling pathways [25], and high expression of integrin a6 was reported to induced AKT and extracellular signal-regulated kinase 1 and 2 (ERK1/2) activation in CCA cells [23]. To verify whether TSPAN1 affects integrin α6β1 signaling, we measured the amplitude of FAK, Src, ERK1/2, PI3K, Akt, p38 and their phosphorylation in CCLP1-TSPAN1 and HCCC9810-shTSPAN1 cells compared with their respective control cells (Fig. 8a). The results showed that p-FAK (Y397), p-Src, p-PI3K, and p-AKT expression changed with TSPAN1 alteration. However, p-FAK (Y925), p-ERK1/2, and p-p38 showed no variation, indicating that TSPAN1 promoted the PI3K/AKT pathway independently. Laminin 5, the ligand of integrin α6β1, was used to activate the integrin α6β1 downstream signaling, and the results showed that CCLP1-TSPAN1 cells exhibited higher activity levels of AKT at different times (Fig. 8b) [26, 27] Taken together, these observations suggested that TSPAN1 promoted the PI3K/AKT pathway by binding with integrin α6β1 in CCA cells.

As activated downstream molecules of AKT, nuclear factor-kB and GSK-3β were investigated, and the results showed that p-GSK-3β was more obviously altered than p-p65, and Snail was changed rather than other EMT-related transcription factors including Slug, Twist, and Zeb1 (Fig. 8c and Additional file 10: Figure S8a) [28, 29]. We found that LY294002 (an inhibitor of PI3K, 20 μM) effectively reversed the AKT pathway (Fig. 8d) and markedly decreased migration and invasion of CCLP1-TSPAN1 cells in vitro (Fig. 8e and Additional file 10: Figure S8b). This observation demonstrated that TSPAN1 was positively correlated with the PI3K/AKT/GSK-3β/Snail pathway.

To further evaluate the role of Snail in the TSPAN1-mediated promotion of CCA cell invasion and metastasis, it was knocked down and overexpressed in CCLP1-TSPAN1 and HCCC9810-shTSPAN1 cells, respectively (lenti-shSnail-1 was chosen for the experiment, Additional file 10: Figure S8c). To confirm these observations in vivo, we performed a lung metastasis assay using bioluminescence imaging with luciferase-expressing cells (*n* = 10/group, Fig. 8f). After the mice were euthanized, the incidence rate of the tumor was recorded, and their lungs were dissected and analyzed using H&E staining (Fig. 8g and Additional file 10: Figure S8d). It was shown that the number of lung metastasis was decreased in CCLP1-TSPAN1 group after expression of shSnail, whereas Snail overexpression significantly increased the metastatic capabilities of HCCC9810-shTSPAN1 cells. These results indicated that Snail was an important downstream effector of TSPAN1-mediated promotion of CCA cell invasiveness and metastasis.

Previous studies have reported that PTEN, a negative regulator of PI3K, could be inhibited by Snail in HCC, pancreatic adenocarcinoma, and Madin Darby canine kidney cells. Thus, we first explored whether the PI3K/AKT/GSK-3β/Snail/PTEN feedback loop existed in CCA cells [26, 30]. Finally, in CCLP1 and HCCC9810 cells, we observed that Snail overexpression decreased PTEN expression, whereas its suppression enhanced PTEN protein and reversed the AKT/GSK-3β pathway activation (Fig. 8h), demonstrating Snail could inhibit PTEN expression in CCA cells. These data proved that TSPAN1 was negatively regulated by miR-194-5p and facilitated PI3K/AKT/GSK-3β/Snail/PTEN feedback loop in CCA pathogenesis (Fig. 8i).

## Discussion

The *TSPAN1* gene is located on chromosome 1p34.1 and encodes the 26 kDa protein of the same name (TSPAN1), which is mainly expressed on the plasma membrane, and on some intracellular vesicles in various tissues [31]. Recent research studies on TSPAN1 have focused on its ability to promote proliferation and migration in cancers. Nevertheless, the mechanism underlying the actions of TSPAN1 are not well understood, and there are limited studies. We first time identify the TSPAN1 function and specific mechanism in human CCA progress.

In the present study, we evaluated TSPAN1 expression in clinical CCA specimens and relevant cell lines, and the results indicated that TSPAN1 was highly expressed at the mRNA and protein levels. The statistical analysis determined that TSPAN1 upregulation was associated with the TNM stage and metastasis. In agreement with the clinicopathological features, our data revealed that TSPAN1 overexpression enhanced tumorigenesis, and as emphasized by research studies, some metastasis assays in vitro and in vivo confirmed that TSPAN1 promoted metastasis in CCA. In the next step, we verified that TSPAN1 induced EMT, and TSPAN1 knockdown resulted in the upregulation of E-cadherin and decrease of N-cadherin and vimentin.

We sought to investigate how miRNA deregulation affects tumors, so we screened deregulated miRNA, which is a contributing element in the high expression of TSPAN1 in CCA. Low expression of miR-194-5p was observed in CCA tissues and miR-194-5p suppressed CCA metastasis in our study. Then, we validated the complementary binding between miR-194-5p and TSPAN1–3′-UTR through the luciferase reporter assay. Most importantly, we proved that a combination of high TSPAN1 expression and low miR-194-5p expression predicts a poor prognosis in CCA patients.

EMT has been shown to significantly accelerate metastasis in epithelium-derived carcinoma including CCA [32, 33]. EMT is a complex, transient, reversible biological process that makes tumors epithelial cells lose polarity, turns adherent phenotypes into mesenchymal forms, and increases cell invasion and migration ability. Alteration of adhesion molecules and the ECM are two key factors in the EMT process [34]. For reference, we paid close attention to the functional characteristics of the TSPAN superfamily of proteins and discovered that they are always associated with adhesion receptors of the integrin family in regulating integrin-dependent cell migration [35]. For example, Herlevsen et al. [36] found that the association of TSPAN8 with α6β4 integrin supported cell motility and liver metastasis formation, and Ai et al. [26] discovered that TSPAN24 interacted with Integrin α6β1 to amplify PI3K/AKT signaling to induce EMT in HCC. Therefore, we focused on several integrins that play stimulatory roles in CCA migration: Utispan et al. [22] reported that integrin α5β1 promoted the invasion of CCA through a PI3K/AKT-dependent pathway. Ding et al. [23] demonstrated that a high level of Integrin a6 expression was associated with a migratory and invasive phenotype of intrahepatic CCA cells, and Patsenker et al. [24] found that aVβ6 integrin is a highly specific IHC marker for CCA. We confirmed α6β1 from these integrins using co-IP and IF techniques to demonstrate its interaction with TSPAN1. We examined pivotal downstream molecules after TSPAN1 transfection using western blotting and discovered that TSPAN1 was involved in the PI3K/AKT pathway. A time-response experiment with Laminin 5 in CCA cells further implied that TSPAN1 amplified PI3K/AKT pathway signaling. Then, we determined whether there was a visible difference in p-GSK-3β and Snail expression. Finally, we found that Snail inhibited PTEN expression in CCLP1 and HCCC9810 cells, indicating PI3K/AKT/GSK-3β/Snail/PTEN feedback loop existed in CCA.

## Conclusions

We provide the first evidence that *TSPAN1* might be a critical oncogene that contributes to CCA growth and metastasis. Upregulation of TSPAN1 is the consequence of miR-194-5p low expression in CCA. Besides, our experiment showed that TSPAN1 interacted with integrin α6β1 to amplify PI3K/AKT/GSK-3β/Snail/PTEN feedback loop to induce EMT in CCA. These findings indicate that TSPAN1 could serve as a prognostic biomarker and therapeutic target for CCA treatment.

## Additional files

Additional file 1: **Table S1.** Target sequences of vshRNAs used in this study. **Table S2.** Primary antibodies for WB, IHC, IF and co-IP used in this study. **Table S3.** Sequence of primers for qRT-PCR. (DOC 86 kb)

Additional file 2: **Figure S1.** TSPAN1 is frequently upregulated in human CCA. The patients with CCA were divided into two groups: the TSPAN1-positive and -negative/low groups. Scale bars: 100× = 100 μm; 200× = 50 μm. (TIF 1280 kb)

Additional file 3: **Table S4.** Univariate and multivariate analyses of factors associated with survival in CCA patients. (DOC 25 kb)

Additional file 4: **Figure S2.** TSPAN1 promotes CCA cell proliferation and tumorigenicity in vitro and in vivo . (a) After lentivirus transfection, TSPAN1 was overexpressed or knocked down in CCA cells. (b) Ki-67 expression proportion in xenograft tissues from CCA cells was counted. Data are means ± SD of three independent experiments. *p < 0.05, **p < 0.01, ***p < 0.001. (TIF 394 kb)

Additional file 5: **Figure S3.** TSPAN1 promotes CCA cell migration and invasion in vitro and in vivo. Uncoated (for migration) transwell assays showed TSPAN1 promoted CCA cell migration. Data are means ± SD of three independent experiments. *p < 0.05, **p < 0.01. (TIF 766 kb)

Additional file 6: **Figure S4.** TSPAN1 induces EMT in CCA. Morphological characteristics of CCLP1 and HCCC9810 after TSPAN1 transfection. (TIF 924 kb)

Additional file 7: **Figure S5.** TSPAN1 is a downstream target of miR-194-5p. (a) Using qRT-PCR method, we analyzed miR-27a, miR-27b, and miR-197-3p in CCA and normal tissues. (b) Relative expression of miR-194-5p in normal liver cell line L02, normal human biliary cell line HIBEpiC and CCA cell lines. (c) After transfection, miR-194-5p was knocked down in Huh28, CCLP, and RBE cells and overexpressed in HCCC9810 and HuCCT1 cells. Data are means ± SD of three independent experiments. *p < 0.05, **p < 0.01, ***p < 0.001. (TIF 383 kb)

Additional file 8: **Figure S6.** MiR-194-5p inhibits CCA metastasis and EMT. (a) Silencing miR-194-5p promoted CCA cells migration, whereas overexpressing miR-194-5p suppressed migration in wound healing assay; representative images were captured at 0 and 24 h. (b) Uncoated (for migration) transwell assays showed miR-194-5p inhibited CCA cell migration. Data are means ± SD of three independent experiments. *p < 0.05, **p < 0.01, ***p < 0.001. (TIF 777 kb)

Additional file 9: **Figure S7.** TSPAN1 interacts with integrin α6β1. TSPAN1 did not interact with integrin α2β1, α5β1, α6β4, and αVβ6. (TIF 284 kb)

Additional file 10: **Figure S8.** TSPAN1 enhances PI3K/AKT/GSK-3β/Snail/PTEN feedback loop. (a) There were no changes in p-p65, Slug, Twist, Zeb1 between CCLP1-TSPAN1, HCCC9810-shTSPAN1 and their control cells. (b) LY294002 markedly decreased migration and invasion of CCLP1-TSPAN1 cells in vitro*.* (c) After transfection, Snail was knocked down in CCLP1 cells and overexpressed in HCCC9810 cells. (d) The incidence of lung metastases observed in the different experimental groups. Data are means ± SD of three independent experiments. *p < 0.05, **p < 0.01. (TIF 363 kb)

## Abbreviations

Akt: Protein kinase B
CCA: Cholangiocarcinoma
EMT: Epithelial-mesenchymal transition
ERK: Extracellular regulated protein kinase
FAK: Focal adhesion kinase
GSK-3β: Glycogen synthase kinase 3β
HCC: Hepatocellular carcinoma
LV: Lentivirus
NF-kB: Nuclear factor-kappaB
PI3K: Phosphatidylinositol-3-kinase
PTEN: Phosphatase and tensin homolog
qRT-PCR: Real-time quantitative reverse transcription-polymerase chain reaction
