# Supplementary material for: Tetraspanin 1 promotes epithelial-to-mesenchymal transition and metastasis of cholangiocarcinoma via PI3K/AKT signaling
Source: J Exp Clin Cancer Res. 2018 Dec 4;37:300. doi: 10.1186/s13046-018-0969-y (PMC6280496; doi:10.1186/s13046-018-0969-y)
Supplement: Supplementary file 1 — Table S1. Target sequences of vshRNAs used in this study. Table S2. Primary antibodies for WB, IHC, IF and co-IP used in this study. Table S3. Sequence of primers for qRT-PCR. (DOC 86 kb) [file 13046_2018_969_MOESM1_ESM.doc]

Table S1. vshRNA Target Sequences

|  | Target sequence | NCBI reference |
| --- | --- | --- |
| TSPAN1 |  |  |
| vshRNA#1 | CATTCTGTTGCAATGACAA | NM_005727 |
| vshRNA#2 | ATCAAGAAAGATTATGGTT | NM_005727 |
| vshRNA#3 | TCTTCAATTTGCTCATCTT | NM_005727 |
| Snail |  |  |
| vshRNA#1 | ACCCCAATCGGAAGCCTAACT | NM_005985 |
| vshRNA#2 | AACAAGGAATACCTCAGCC | NM_005985 |
| vshRNA#3 | GCCTAACTACAGCGAGCTG | NM_005985 |

Table S2. Primary antibodies for WB, IHC, IF and coIP

| Antibody | Concentration  for WB | Concentration  for IHC | Concentration  for IF | Concentration  for co-IP | Specificity | Company |
| --- | --- | --- | --- | --- | --- | --- |
| TSPAN1 | 1:1000 | 1:200 | 1:200 | 5μg/ml | Rabbit polyclonal  Mouse monoclonal | Abcam  Santa Cruz |
| E-cadherin | 1:1000 |  | 1:100 |  | Mouse monoclonal | Abcam |
| N-cadherin | 1:1000 |  |  |  | Rabbit polyclonal | Abcam |
| Vimentin | 1:2000 |  | 1:200 |  | Rabbit polyclonal | Abcam |
| ITGA2 | 1:1000 |  |  |  | Rabbit monoclonal | Abcam |
| ITGA5 | 1:2000 |  |  |  | Rabbit monoclonal | Abcam |
| ITGA6 | 1:1000 |  | 1:100 | 4μg/ml | Rabbit polyclonal  Rat monoclonal | Abcam  Abcam |
| ITGAV | 1:1000 |  |  |  | Rabbit monoclonal | Abcam |
| ITGB1 | 1:1000 |  |  |  | Rabbit monoclonal | Abcam |
| ITGB4 | 1:1000 |  |  |  | Rabbit monoclonal | Abcam |
| ITGB6 | 1:1000 |  |  |  | Rabbit monoclonal | Abcam |
| FAK | 1:1000 |  |  |  | Rabbit monoclonal | CST |
| p-FAK(Y397) | 1:1000 |  |  |  | Rabbit monoclonal | CST |
| p-FAK(Y925) | 1:1000 |  |  |  | Rabbit polyclonal | CST |
| Src | 1:1000 |  |  |  | Rabbit monoclonal | CST |
| p-Src | 1:500 |  |  |  | Rabbit monoclonal | CST |
| PI3K | 1:500 |  |  |  | Rabbit monoclonal | CST |
| p-PI3K | 1:500 |  |  |  | Rabbit polyclonal | CST |
| AKT | 1:1000 |  |  |  | Rabbit monoclonal | CST |
| p-AKT | 1:1000 |  |  |  | Rabbit monoclonal | CST |
| ERK1/2 | 1:1000 |  |  |  | Rabbit monoclonal | CST |
| p-ERK1/2 | 1:1000 |  |  |  | Rabbit monoclonal | CST |
| p38 | 1:1000 |  |  |  | Rabbit monoclonal | CST |
| p-p38 | 1:1000 |  |  |  | Rabbit monoclonal | CST |
| GSK-3β | 1:2000 |  |  |  | Rabbit polyclonal | Abcam |
| p-GSK-3β | 1:2000 |  |  |  | Rabbit polyclonal | Abcam |
| p65 | 1:1000 |  |  |  | Rabbit monoclonal | CST |
| p-p65 | 1:1000 |  |  |  | Mouse monoclona | CST |
| Snail | 1:1000 |  |  |  | Goat polyclonal | Abcam |
| Slug | 1:1000 |  |  |  | Rabbit polyclonal | Abcam |
| TWIST | 1:1000 |  |  |  | Mouse monoclonal | Abcam |
| ZEB1 | 1:1000 |  |  |  | Rabbit monoclonal | Abcam |
| PTEN | 1:1000 |  |  |  | Rabbit monoclonal | Abcam |

Table S3. Sequence of Primers for qRT-PCR

| Primers |  | Sequences (5’------3’) |
| --- | --- | --- |
| TSPAN1 | Forward： | 5’-CGTTGTGGTCTTTGCTCTTG-3’ |
|  | Reverse： | 5’-TTCTTGATGGCAGGCACTAC-3’. |
| E-cadherin | Forward： | 5’-CTGAGAACGAGGCTAACG-3’ |
|  | Reverse： | 5’-TTCACATCCAGCACATCC-3’ |
| N-cadherin | Forward： | 5’-GTAGCTAATCTAACCTGATAAGG-3’ |
|  | Reverse： | 5’-TTGGTTTGACCACGGTGACTA-3’ |
| Vimentin | Forward： | 5’-TTGAACGCAAAGTGGAATC-3’ |
|  | Reverse： | 5’-AGGTCAGGCTTGGAAACA-3’ |
| GAPDH | Forward： | 5’-GCACCGTCAAGGCTGAGAAC-3’ |
|  | Reverse： | 5’-TGGTGAAGACGCCAGTGGA-3’ |
| hsa-miR-194-5p TaqMan™ MicroRNA (Life Technologies, 002355, Cat#: 4427975) | | |
