# Supplementary figures and images for: Tetraspanin 1 promotes epithelial-to-mesenchymal transition and metastasis of cholangiocarcinoma via PI3K/AKT signaling
Source: J Exp Clin Cancer Res. 2018 Dec 4;37:300. doi: 10.1186/s13046-018-0969-y (PMC6280496; doi:10.1186/s13046-018-0969-y)

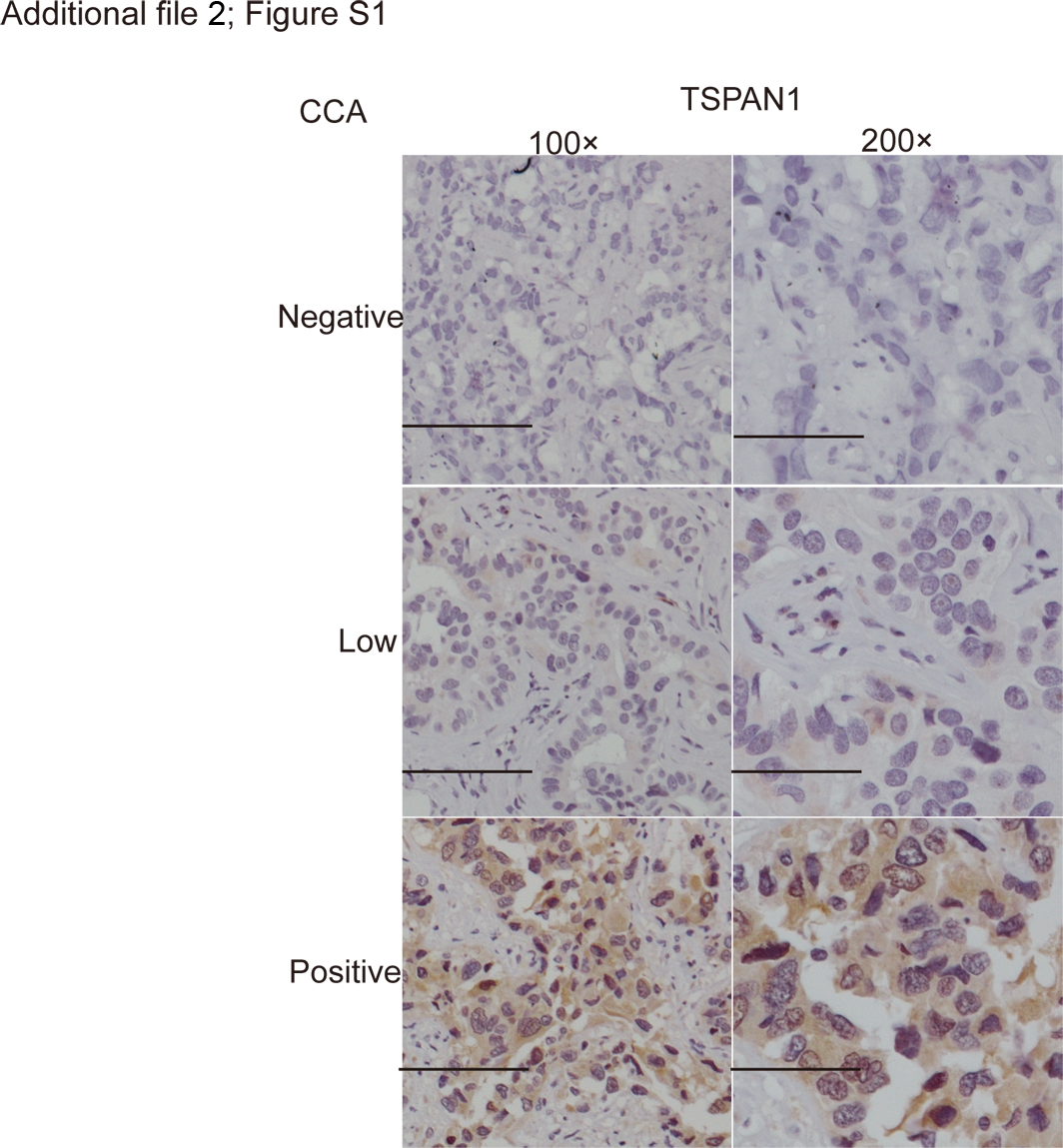

Supplement: Supplementary file 2 — Figure S1. TSPAN1 is frequently upregulated in human CCA. The patients with CCA were divided into two groups: the TSPAN1-positive and -negative/low groups. Scale bars: 100× = 100 μm; 200× = 50 μm. (TIF 1280 kb) [file 13046_2018_969_MOESM2_ESM.tif]

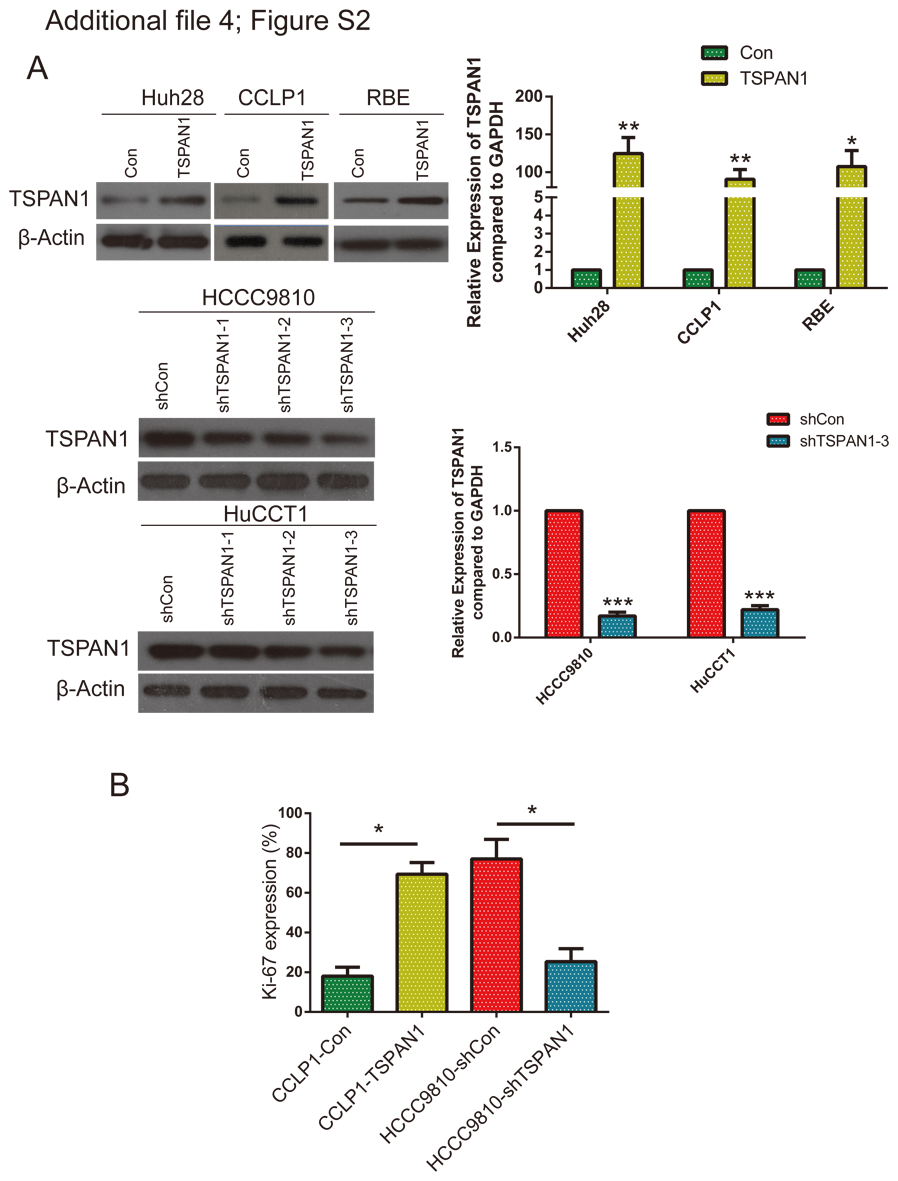

Supplement: Supplementary file 4 — Figure S2. TSPAN1 promotes CCA cell proliferation and tumorigenicity in vitro and in vivo . (a) After lentivirus transfection, TSPAN1 was overexpressed or knocked down in CCA cells. (b) Ki-67 expression proportion in xenograft tissues from CCA cells was counted. Data are means ± SD of three independent experiments. *p < 0.05, **p < 0.01, ***p < 0.001. (TIF 394 kb) [file 13046_2018_969_MOESM4_ESM.tif]

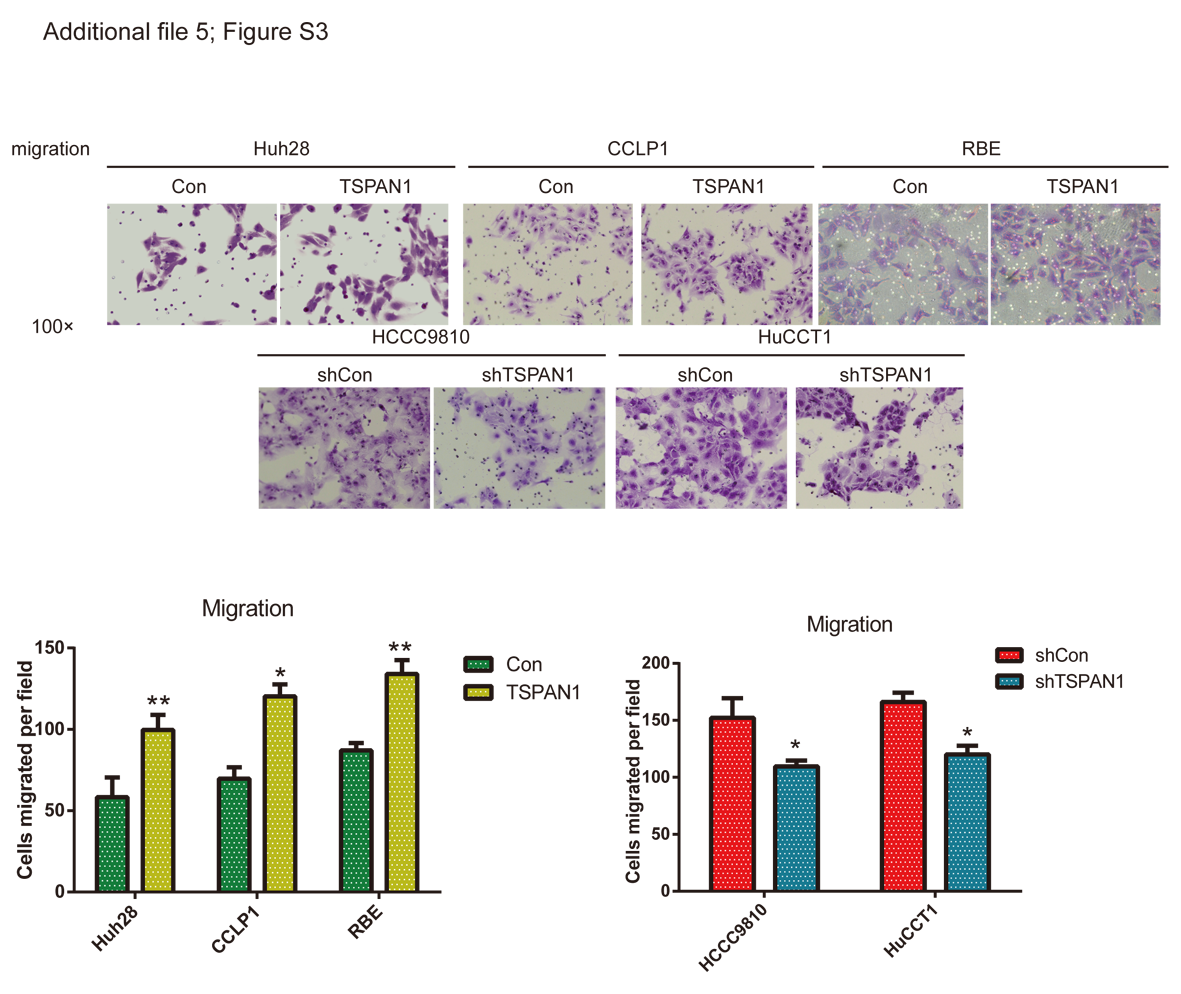

Supplement: Supplementary file 5 — Figure S3. TSPAN1 promotes CCA cell migration and invasion in vitro and in vivo. Uncoated (for migration) transwell assays showed TSPAN1 promoted CCA cell migration. Data are means ± SD of three independent experiments. *p < 0.05, **p < 0.01. (TIF 766 kb) [file 13046_2018_969_MOESM5_ESM.tif]

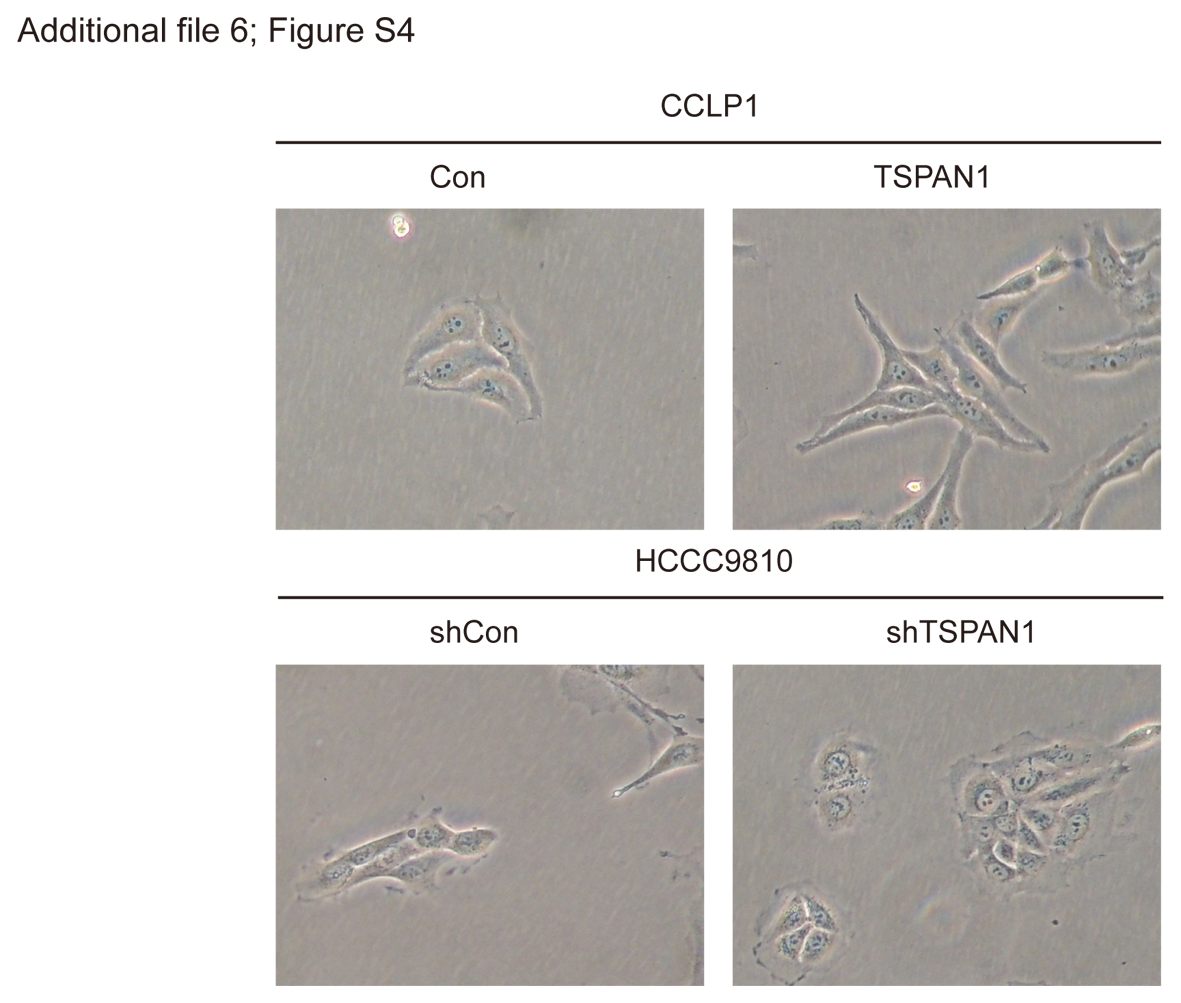

Supplement: Supplementary file 6 — Figure S4. TSPAN1 induces EMT in CCA. Morphological characteristics of CCLP1 and HCCC9810 after TSPAN1 transfection. (TIF 924 kb) [file 13046_2018_969_MOESM6_ESM.tif]

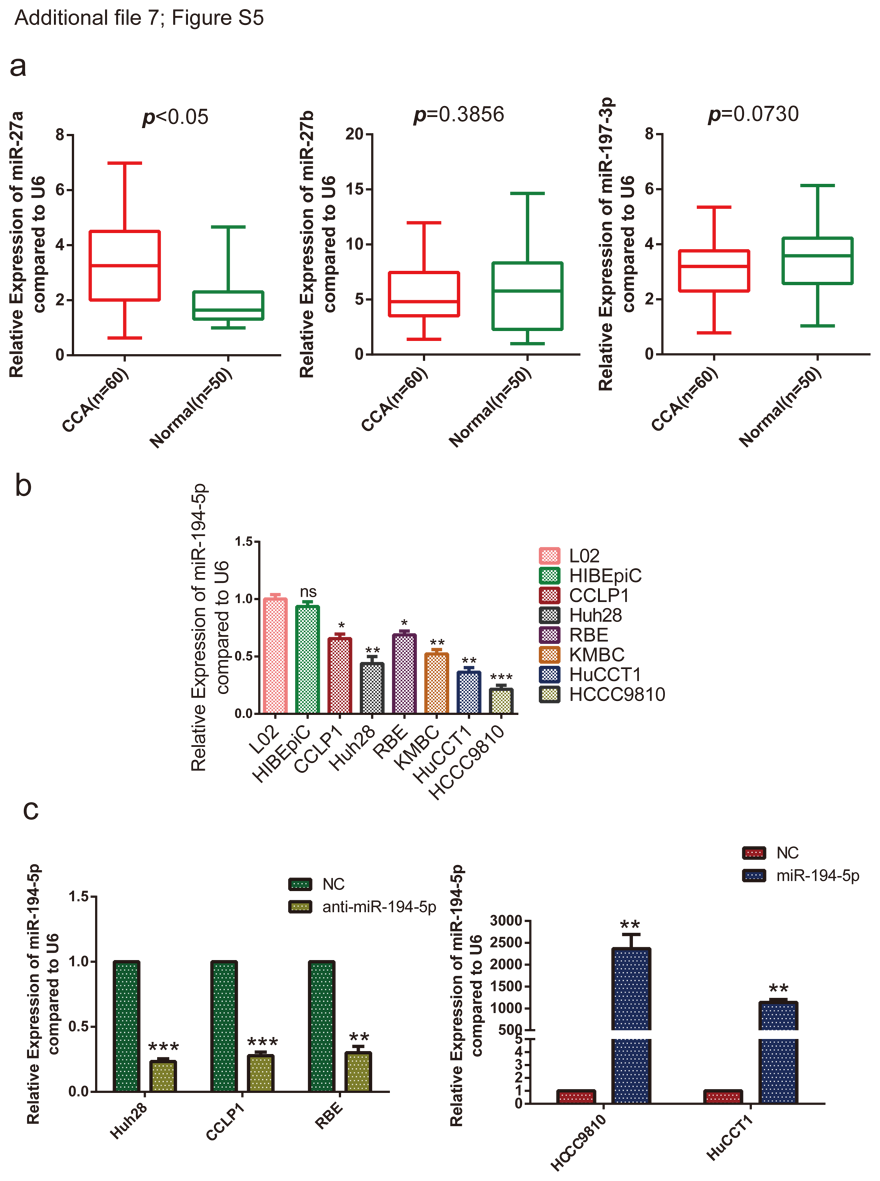

Supplement: Supplementary file 7 — Figure S5. TSPAN1 is a downstream target of miR-194-5p. (a) Using qRT-PCR method, we analyzed miR-27a, miR-27b, and miR-197-3p in CCA and normal tissues. (b) Relative expression of miR-194-5p in normal liver cell line L02, normal human biliary cell line HIBEpiC and CCA cell lines. (c) After transfection, miR-194-5p was knocked down in Huh28, CCLP, and RBE cells and overexpressed in HCCC9810 and HuCCT1 cells. Data are means ± SD of three independent experiments. *p < 0.05, **p < 0.01, ***p < 0.001. (TIF 383 kb) [file 13046_2018_969_MOESM7_ESM.tif]

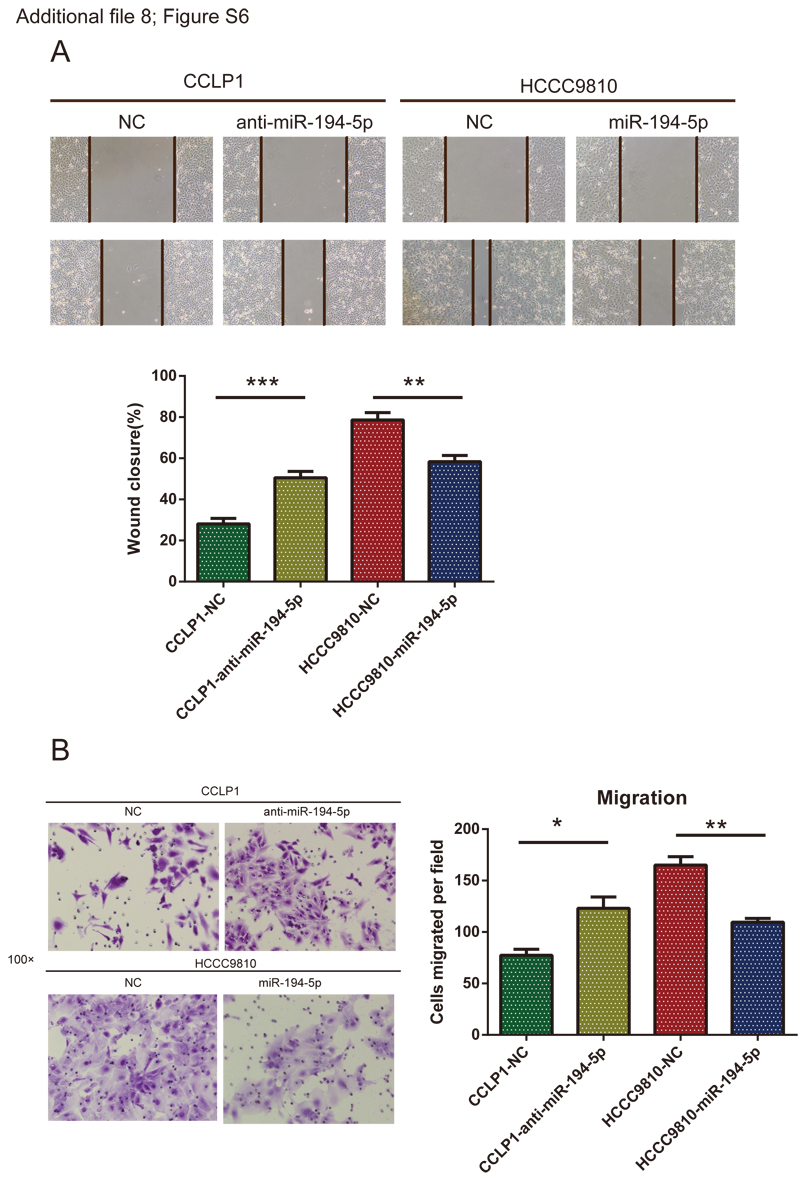

Supplement: Supplementary file 8 — Figure S6. MiR-194-5p inhibits CCA metastasis and EMT. (a) Silencing miR-194-5p promoted CCA cells migration, whereas overexpressing miR-194-5p suppressed migration in wound healing assay; representative images were captured at 0 and 24 h. (b) Uncoated (for migration) transwell assays showed miR-194-5p inhibited CCA cell migration. Data are means ± SD of three independent experiments. *p < 0.05, **p < 0.01, ***p < 0.001. (TIF 777 kb) [file 13046_2018_969_MOESM8_ESM.tif]

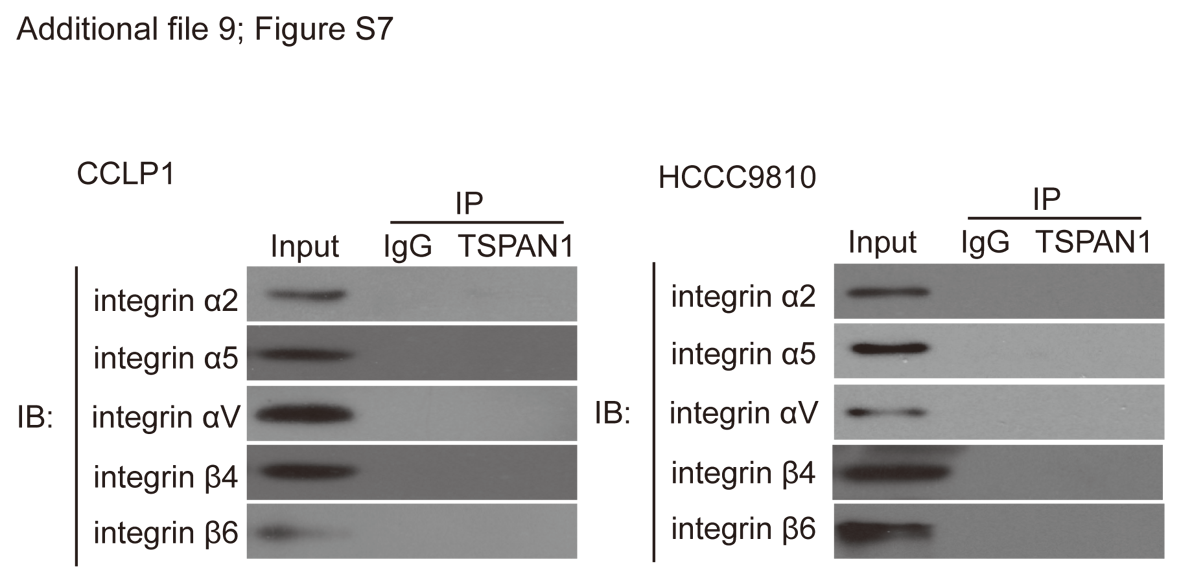

Supplement: Supplementary file 9 — Figure S7. TSPAN1 interacts with integrin α6β1. TSPAN1 did not interact with integrin α2β1, α5β1, α6β4, and αVβ6. (TIF 284 kb) [file 13046_2018_969_MOESM9_ESM.tif]

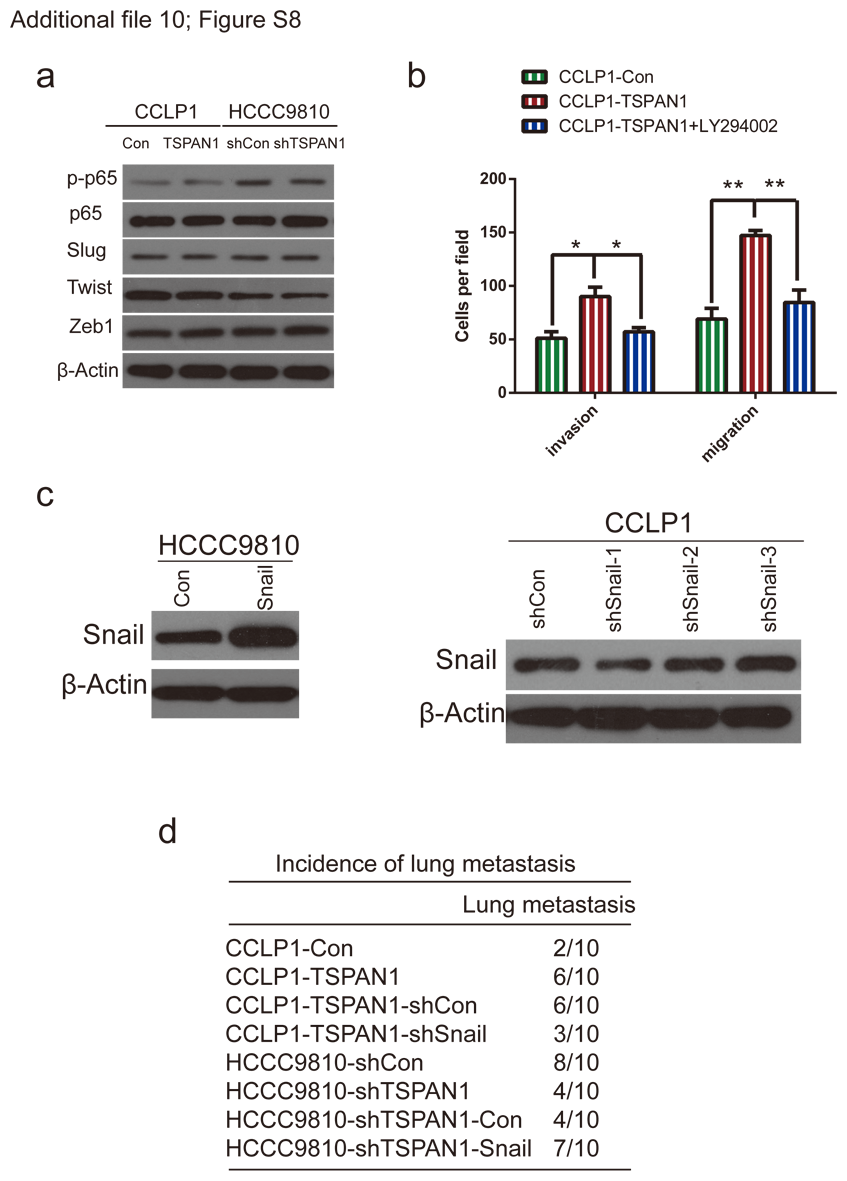

Supplement: Supplementary file 10 — Figure S8. TSPAN1 enhances PI3K/AKT/GSK-3β/Snail/PTEN feedback loop. (a) There were no changes in p-p65, Slug, Twist, Zeb1 between CCLP1-TSPAN1, HCCC9810-shTSPAN1 and their control cells. (b) LY294002 markedly decreased migration and invasion of CCLP1-TSPAN1 cells in vitro. (c) After transfection, Snail was knocked down in CCLP1 cells and overexpressed in HCCC9810 cells. (d) The incidence of lung metastases observed in the different experimental groups. Data are means ± SD of three independent experiments. *p < 0.05, **p < 0.01. (TIF 363 kb) [file 13046_2018_969_MOESM10_ESM.tif]
