# Supplementary material for: Tetraspanin 1 promotes epithelial-to-mesenchymal transition and metastasis of cholangiocarcinoma via PI3K/AKT signaling
Source: J Exp Clin Cancer Res. 2018 Dec 4;37:300. doi: 10.1186/s13046-018-0969-y (PMC6280496; doi:10.1186/s13046-018-0969-y)
Supplement: Supplementary file 3 — Table S4. Univariate and multivariate analyses of factors associated with survival in CCA patients. (DOC 25 kb) [file 13046_2018_969_MOESM3_ESM.doc]

Table . Univariate and multivariate analyses of factors associated with survival in CCA patients.

Overall Survival

|  |  | Multivariate analysis | | |
| --- | --- | --- | --- | --- |
| Factors | Univariate P | HR | 95% CI | P |
| Age (< 60 vs ≥60 years) | 0.962 | － | － | － |
| Gender (female vs male) | 0.910 | － | － | － |
| Histological differentiation (P vs M/W) | 0.331 | － | － | － |
| CA19-9 (U/ml) (≤37 vs > 37) | 0.284 | － | － | － |
| TNM stage (I/II vs III/IV) | 0.049 | 1.559 | 0.862-2.819 | 0.142 |
| pN (lymph node metastasis) (pN0 vs pN1) | 0.054 | － | － | － |
| Distant metastasis (M0 vs M1) | 0.063 | － | － | － |
| TSPAN1 ( negative/low vs positive) | 0.028 | 2.051 | 1.080-3.898 | 0.028 |
